# Decoding the biological information contained in two ancient Slavonic parchment codices: an added historical value

**DOI:** 10.1111/1462-2920.15064

**Published:** 2020-05-29

**Authors:** Guadalupe Piñar, Hakim Tafer, Manfred Schreiner, Heinz Miklas, Katja Sterflinger

**Affiliations:** ^1^ Institute of Microbiology and Microbial Biotechnology, Department of Biotechnology University of Natural Resources and Life Sciences, Muthgasse 11, A‐1190 Vienna Austria; ^2^ Institute of Science and Technology in Art (ISTA) Academy of Fine Arts Vienna Schillerplatz 3, A‐1010 Vienna Austria; ^3^ Department of Slavonic Studies University of Vienna Spitalgasse 2‐4, Hof 3, A‐1090 Vienna Austria

## Abstract

This study provides an example in the emerging field of biocodicology showing how metagenomics can help answer relevant questions that may contribute to a better understanding of the history of ancient manuscripts. To this end, two Slavonic codices dating from the 11th century were investigated through shotgun metagenomics. Endogenous DNA enabled to infer the animal origin of the skins used in the manufacture of the two codices, while nucleic sequences recovered from viruses were investigated for the first time in this material, opening up new possibilities in the field of biocodicology.

In addition, the microbiomes colonizing the surface of the parchments served to determine their conservation status and their latent risk of deterioration. The saline environment provided by the parchments selected halophilic and halotolerant microorganisms, which are known to be responsible for the biodegradation of parchment. Species of *Nocardiopsis*, *Gracilibacillus* and *Saccharopolyspora*, but also members of the *Aspergillaceae* family were detected in this study, all possessing enzymatic capabilities for the biodeterioration of this material. Finally, a relative abundance of microorganisms originating from the human skin microbiome were identified, most probably related to the intensive manipulation of the manuscripts throughout the centuries, which should be taken with caution as they can be potential pathogens.

## Introduction

Biocodicology is an emerging field that studies the biological information stored in ancient manuscripts and is currently revolutionizing the field of codicology (Fiddyment *et al*., 2019) by incorporating high‐throughput molecular techniques such as proteomics (Fiddyment *et al*., 2015), genomics and metagenomics (Teasdale *et al*., 2015, 2017). These technologies make it possible to extract the biological information stored for centuries in ancient manuscripts, especially in parchment objects.

Objects preserved in museums and cultural archives offer the potential not only to extract preserved host DNA but also DNA from associated pathogens or even microbiomes (Green and Speller, 2017). Several studies have highlighted the suitability of parchment as a good reservoir of ancient DNA (Bower *et al*., 2010; Campana *et al*., 2010; Teasdale *et al*., 2015; Green and Speller, 2017). The DNA can be primarily preserved in parchments as a consequence of the manufacturing process. Parchments are elaborated from skins of domestic animals, mainly bovine, ovine and goat that go through a process of washing and salt‐curing, followed by depilation, stretching, drying, scraping and pouncing (Bower *et al*., 2010; Campana *et al*., 2010). This manufacturing process produces a durable substrate, which can survive undamaged for centuries. In addition, and because of their legal, religious or historical value, many of these manuscripts have been carefully preserved in numerous libraries and archives (Teasdale *et al*., 2015) and represent not only an irreplaceable documentary record but also a remarkable reservoir of biological information.

The application of molecular techniques to recover the biological information contained in manuscripts made of parchment has been going on for several years (Bower *et al*., 2010). Nevertheless, molecular methods are not free of biases and present limitations related mainly to the destructive sampling of the documents to obtain sufficient quantities of starting material for the recovery of genetic material as well as to the intrinsic limitations of the methodologies used prior Next generation sequencing (NGS) approaches were available. However, the need manifested in museums and archives to preserve the integrity of unique objects led to an important development of non‐destructive and non‐invasive sampling methodologies as well as extraction techniques (Wandeler *et al*., 2007; Cappitelli *et al*., 2010; Pinzari *et al*., 2011; Lech, 2016a). Numerous studies have thus highlighted the possibilities offered by genetic analysis on parchment (Bower *et al*., 2010). Some of them have focused on the material itself to achieve the species identification of the animal skins used in the manufacture of parchment (Bower *et al*., 2010; Pangallo *et al*., 2010). In turn, other studies have focused on a practical conservation purpose and have conducted extensive microbiological and environmental research on manuscripts to identify the risk of potential harm exerted by these factors (Sterflinger and Pinzari, 2012; Piñar *et al*., 2015a, 2015b; Lech, 2016b) and to develop restoration methods (Vadrucci *et al*., 2019).

Nowadays, high‐throughput molecular methods enable the extraction and the analysis of the smallest traces of DNA from ancient objects and by the help of bioinformatics and international databases, to assign single molecules of DNA to certain animals, plants, bacteria, fungi or even to humans. The compilation of these data results in an individual microbiome or ‘biological pedigree’ (Piñar *et al*., 2019, 2020), which can be considered as an historical added value. The recovered biological information offers new possibilities to answer relevant questions related to valuable objects made of parchment, such as the book making process, the skin/animal type used to produce the parchment, its conservation status and its historical use (Teasdale *et al*., 2015, 2017; Migliore *et al*., 2017, 2019; Fiddyment *et al*., 2019).

**Fig 1:**
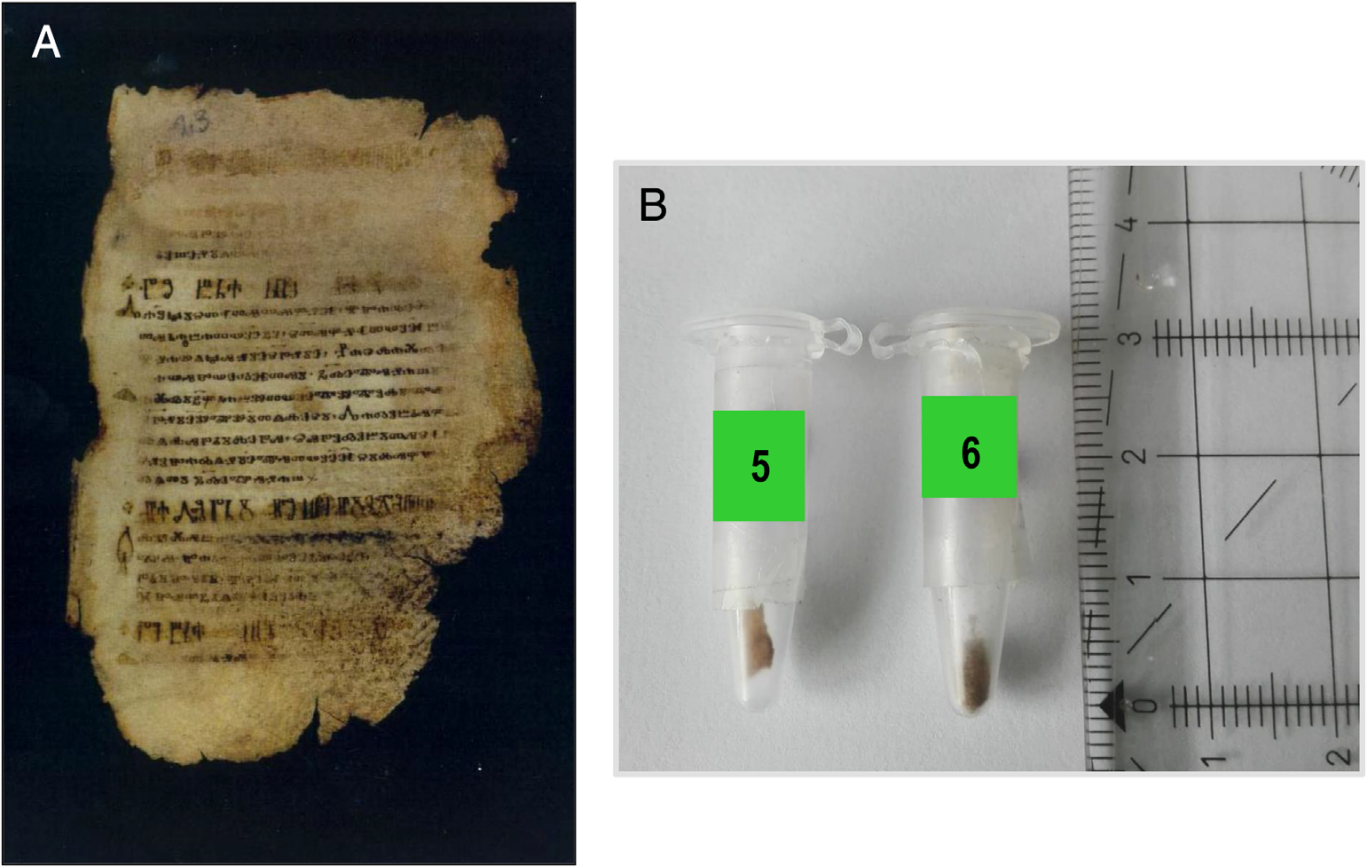
*Liturgiarium Sinaiticum*. A. Detail of a folio. B. sample 5 (Cod. Sin. Slav. 5 N, Folio 3) and sample 6 (Cod. Sin. Slav. 5 N, Fragment EDV 68) obtained from detached fragments of about 2 mm^2^ × 5 mm^2^ each.

**Fig 2:**
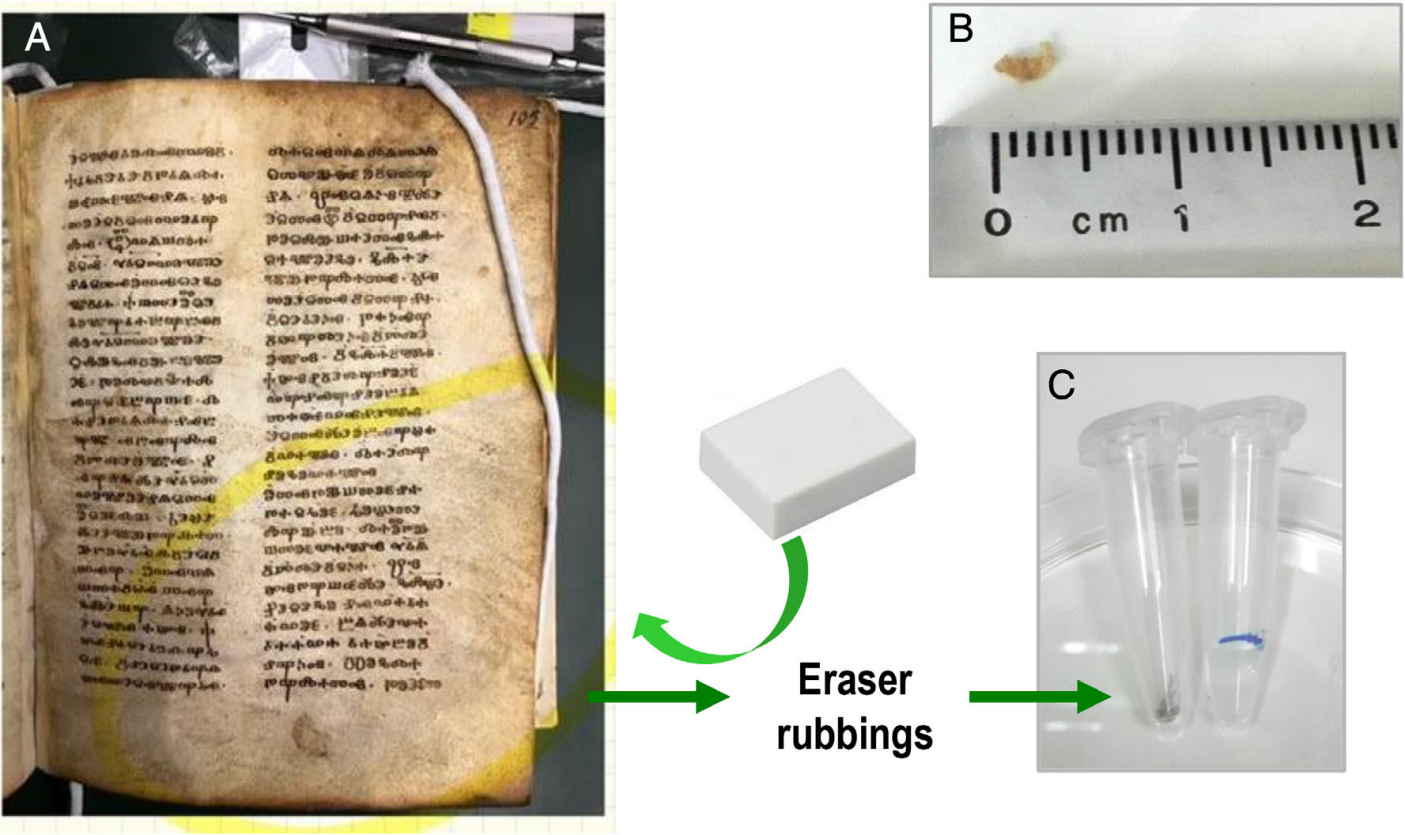
Codex *Assemanianus*. A. Detail of a folio. B. Sample P (Vat‐Slav‐3P) obtained from a detached fragment of about 1 mm^2^ × 4 mm^2^. C. sample R (Vat‐Slav‐Rb) taken with an eraser by rubbing the surface of the parchment and collecting the fragments detached from it in a sterile assay tube.

In this study, we have unified the advantages of extracting total DNA from parchments in order to recover ancient and modern DNA and thus obtain maximum biological information. The retrieved information has been used to answer two main questions: First, to infer the animal origin of the investigated parchments and second, to elucidate the microbiome that colonizes these valuable objects for conservation purposes. To this end, two ancient Slavonic codices, namely the *Liturgiarium Sinaiticum* (commonly *Missale Sinaiticum*) and the Codex *Assemanianus* (Figs. 1 and 2) were investigated by shotgun metagenomic analyses using the Illumina sequencing HiSeq platform.

## Results

### Metagenomic analyses

Metagenomic analyses were performed on two samples obtained from the ‘*Liturgiarium* (*Missale*) *Sinaiticum*’ (both small detached pieces of the codex, Fig. 1) as well as on two samples obtained from the Codex *Assemanianus* (one was a detached piece of the codex and the second sample was taken with an eraser, Fig. 2). In this study, we demonstrate the success in the extraction of DNA from ancient parchment manuscripts, not only from very small pieces of few millimetres but also from eraser fragments detached after rubbing the surface of the parchment (see the Experimental Procedures section). We used a combination of two commercial kits taking advantage of a silica membrane‐based purification of very short fragments of DNA (~70 bp), which is the expected size of ancient DNA. Furthermore, this approach enabled the elution in very small volumes (as little as 10 μl) allowing the concentration of very low DNA yield, as it was the case of the DNA extracted from these ancient and tiny parchment samples. The DNA extracted from both codices ranged from 0.15 to 2.80 ng/μl. The resulting DNA extracts obtained from all four samples were subjected to library preparation to perform shotgun sequencing using the Illumina sequencing platform. The sequencing details are given in Table 1. Data allowed the identification of the animal skin used for the manufacturing of the different parchments as well as to infer the microbiome colonizing their surfaces.

**Table 1:** Sequencing and trimming statistics for the data sets derived from the Codex *Liturgiariu m Sinaiticum* and Codex *Assemanianus* after removing reads containing N and with at a least 25 nucleotides.

	Codex *Liturgiarium Sinaiticum*		Codex *Assemanianus*	
	Sample 5	Sample 6	Sample P	Sample R
Total reads processed	43 150 589	43 969 902	48 628 371	50 416 907
Reads with adapters	25 138 198 (58.3%)	17 430 739 (39.6%)	37 539 990 (77.2%)	42 380,856 (84.1%)
Reads too short	438 982 (1.0%)	843 127 (1.9%)	2 262 026 (4.7%)	11 406 545 (22.6%)
Reads written (passing filters)	42 479 876 (98.4%)	42 864 968 (97.5%)	46 133 414 (94.9%)	38 806 294 (77.0%)
Total basepairs processed	4 315 058 900 bp	4 396 990 200 bp	4 862 837 100 bp	5 041 690 700 bp
Total written (filtered)	2 966 559 125 bp (68.7%)	2 764 309 577 bp (62.9%)	2 924 139 804 bp (60.1%)	2 251 434 123 bp (44.7%)

### Animal species identification of parchments

To infer the animal origin of the animal skins used in the manufacturing of the investigated parchments, the obtained sequencing data were mapped against the goat (ARS1), cow (UMD 3.1.1), sheep (Oar_V4), pig (Sscrofa 11.1) and human genomes (GRCh38). Our results indicated that the samples obtained from the two different folios of the *Liturgiarium Sinaiticum* affiliated with two different animals. Sample 5 showed the highest number of sequences affiliating with sheep, while sample 6 showed the highest number of sequences matching with calf (Table 2). The two samples (samples P and R) derived from the Codex *Assemanianus* showed that the largest number of sequences were affiliated with sheep, indicating that both originated from this animal (Table 2). Some reads mapped against other mammal genomes, but in a lower number, what might be explained by errors due to aDNA degradation and to the animal skin processing steps leading to the manufactured parchment. It is important to note that in the four samples investigated the highest number of reads compared with the abovementioned databases showed to be related to humans, which may be related to the intensive handling of the manuscripts over the centuries.

**Table 2:** Animal species identified in the Codex *Liturgiarium Sinaiticum* and Codex *Assemanianus*, showing the total numbers of reads as well as the relative proportion of the total sequences (%) affiliating with each species.

	Codex *Liturgiarium Sinaiticum*		Codex *Assemanianus*	
Species	Reads sample 5	Reads sample 6	Reads sample P	Reads sample R
*Homo sapiens*	208 416 (0.5%)	64 370 (0.15%)	7 852 951 (17%)	143 268 (0.4%)
*Ovis aries*	**49 223 (0.12%)**	483 (0.001%)	**55 146 (0.12%)**	**24 465 (0.06%)**
*Bos taurus*	15 204 (0.035%)	**4630 (0.01%)**	13 544 (0.03%)	19 958 (0.05%)
*Capra hircus*	8803 (0.02%)	174 (0.0004%)	11 576 (0.025%)	3972 (0.01%)
*Sus scrofa*	4615 (0.01%)	945 (0.002%)	19 598 (0.04%)	3698 (0.01%)

Bold marked: indicate the highest number of reads related to a specific animal, besides humans in each sample.

### Microbiomes

**Fig 3:**
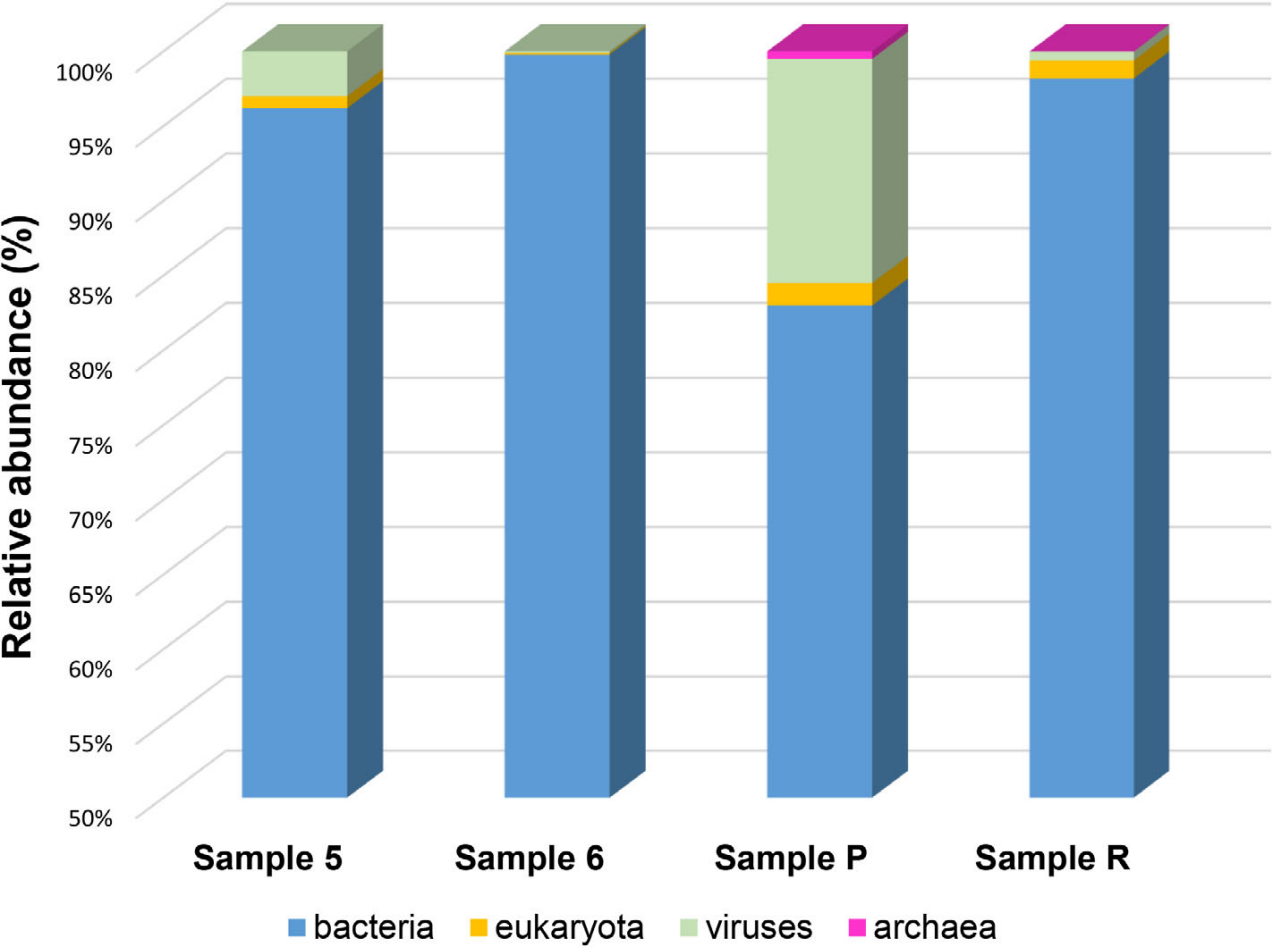
Relative abundance of eukaryotes, bacteria, archaea and viruses in the microbiomes of all four investigated samples. Samples 5 and 6 (*Liturgiarium Sinaiticum*) and samples P and R (Codex *Assemanianus*).

The strategy followed in this study performing shotgun libraries, without any step of amplification of specific target regions, enabled to infer the true proportions of the three domains of life, namely eukaryotes, bacteria and archaea, in addition of viruses. The results indicated that the microbiomes in the four samples consisted mainly of bacteria (Fig. 3), which represented 96% and 99.8% of all sequences in samples 5 and 6, respectively, and 83% and 98.2% in samples P and R respectively. In contrast, the proportion of eukaryotes showed to be very low, representing 0.8%, 0.1%, 1.5% and 1.2% in samples 5, 6, P and R respectively (Fig. 3). Surprisingly, the relative abundance of viruses proved to be relevant in samples 5 (3% of total sequences) and P (15%), while in samples 6 (0.1%) and R (0.6%), they were less dominant (Fig. 3). Archaea were identified only in samples derived from the Codex *Assemanianus*, representing 0.5% of the total sequences in sample P and being almost negligible (0.01%) in sample R (Fig. 3).

#### Bacteria

**Fig 4:**
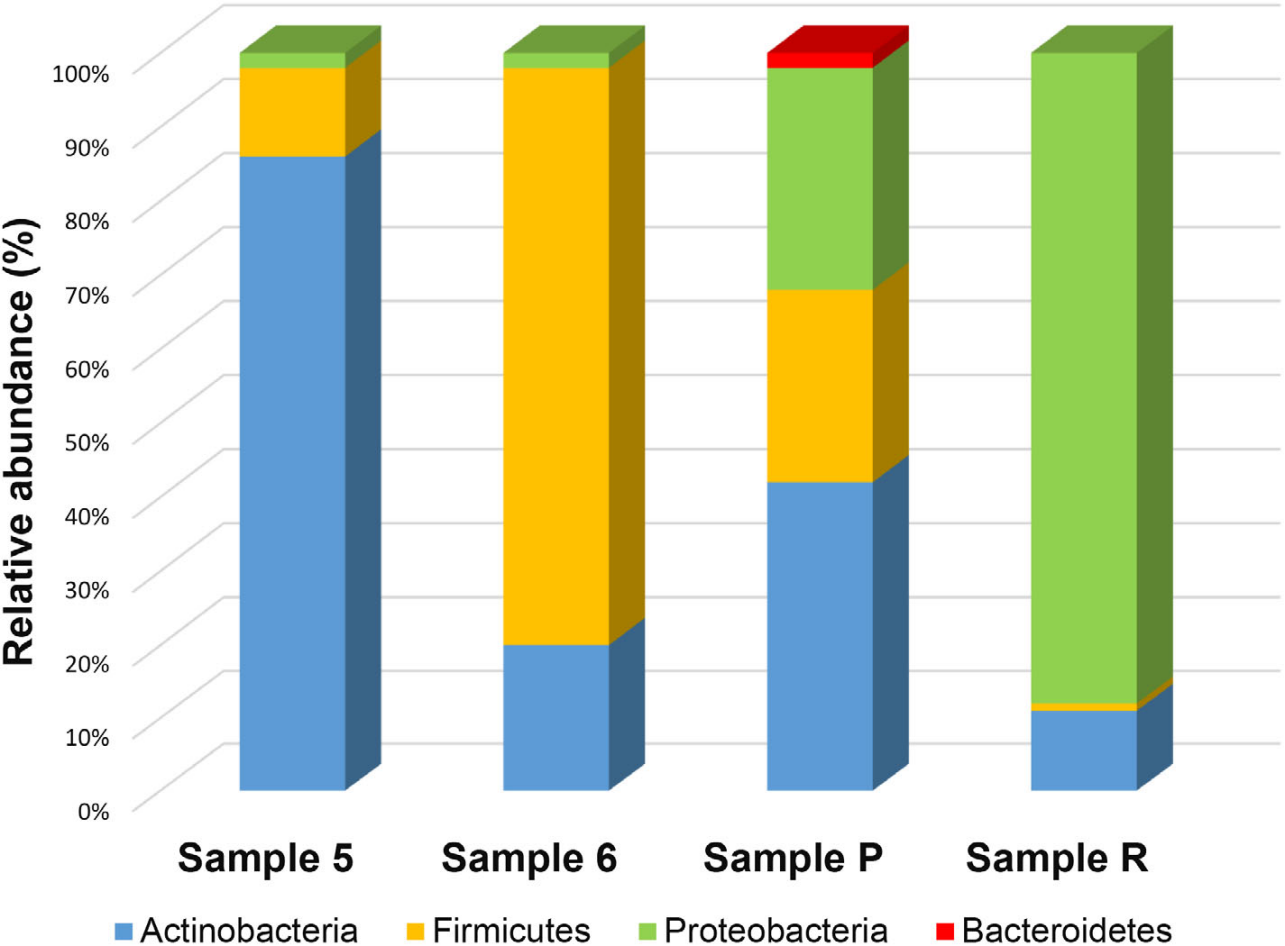
Relative abundance of the bacterial communities of all four investigated samples at the phylum level. Samples 5 and 6 (*Liturgiarium Sinaiticum*) and samples P and R (Codex *Assemanianus*).

**Fig 5:**
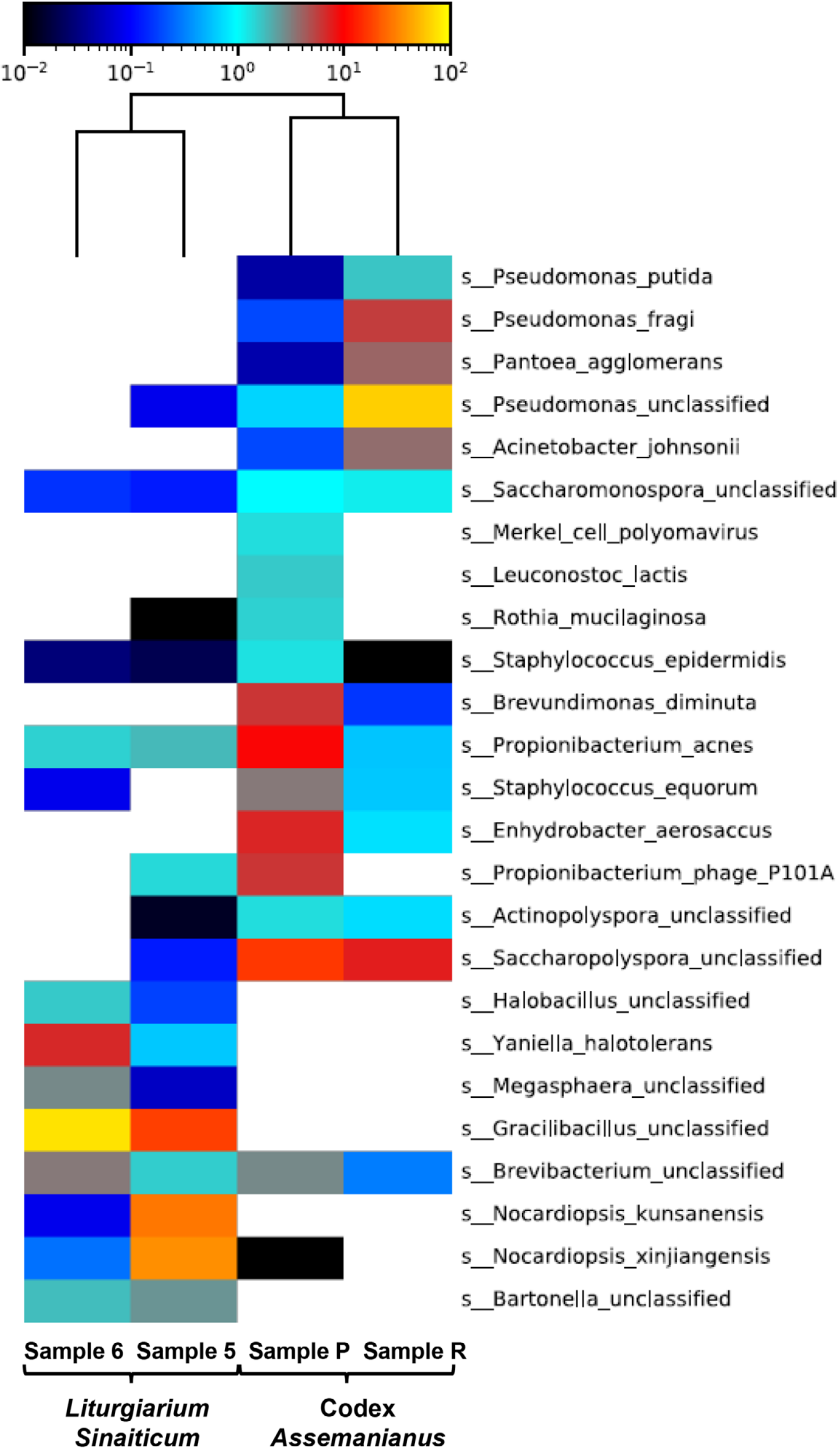
A heatmap reporting only the 25 most abundant clades in all four investigated samples, according to the 90th percentile of the abundances in each clade.

The bacterial communities of the four samples showed similar low diversity, with three shared phyla, namely *Actinobacteria*, *Firmicutes* and *Proteobacteria*, while the phylum *Bacteroidetes* showed to be present only in sample P (2% of total bacteria in this sample). However, important differences were observed among all the samples analysed with respect to the relative abundance of the different phyla (Fig. 4) and the distribution of the most dominant species (Fig. 5). The phylum *Actinobacteria* was dominant in sample 5 (86% of bacteria) and P (42%) but represented only 20% and 11% of bacteria in samples 6 and R, respectively. The phylum *Firmicutes* was dominant only in sample 6, comprising 78% of all bacteria in this sample and accounted for 12%, 26% and 1% of bacteria for samples 5, P and R, respectively. The relative abundance of *Proteobacteria* showed to be identical for the two samples derived from the *Liturgiarium Sinaiticum*, accounting for 2% of bacteria, but it was much higher in samples derived from the Codex *Assemanianus*, representing 30% and 88% of bacteria in samples P and R, respectively.

#### Liturgiarium Sinaiticum

As mentioned above, the bacterial community in sample 5 was dominated by members of the *Actinobacteria* (86% bacteria, Supporting Information Fig. S1A). Members of this phylum grouped into the genera *Nocardiopsis* (79%), including exclusively the species *N. xinjiangensis* and *N. kunsanensis* (Fig. 5) and *Propionibacterium* (2%) with the species *P. acnes* and *P. granulosum* as well as members of the family *Thermomonosporaceae* (2%), being not possible to infer lower taxonomic levels inside this family. In addition, members of the genera *Brevibacterium* and *Yaniella* were detected in lower proportions (0.9% and 0.7% of bacteria, respectively), with sequences affiliating with *Brevibacterium sp*. and *Yaniella halotolerans* (Fig. 5). In contrast, members of this phylum accounted only for 20% of bacteria in sample 6 (Supporting Information Fig. S1B), consisting mainly of species of the genera *Yaniella* (12%), *Propionibacterium* (3%) and *Brevibacterium* (3%), with the identified species *Y. halotolerans* and *P. acnes*.

The phylum *Firmicutes* was dominant in sample 6 (78% bacteria) with unclassified species of the genera *Gracilibacillus* (71%), *Halobacillus* (1%) and *Megasphaera* (3%). This phylum showed a lower proportion in sample 5 (12% of bacteria) but as observed in sample 6, it comprised mainly species of the genus *Gracilibacillus* (11%, Supporting Information Fig. S1).

The relative abundance of *Proteobacteria* showed to be similar in the two samples of this codex (2%) and comprised unclassified species of the genus *Bartonella* (Fig. 5 and Supporting Information Fig. S1).

#### Codex Assemanianus

The bacterial community of the two samples obtained from this codex showed significant differences in the relative proportions of the different phylogenetic groups, but also some interesting similarities (Fig. 5 and Supporting Information Fig. S2). Sample P showed the highest diversity among all the samples analysed with a dominance of *Actinobacteria* (42%). Members of this phylum affiliated mainly with species of the genera *Propionibacterium* (14%), with the species *P. acnes*, and *Saccharopolyspora* (12%). In addition, other identified *Actinobacteria* were related to species of the genera *Corynebacterium*, *Brevibacterium* and *Rothia* (each accounting for 3% of bacteria) as well as to members of the family *Dermabacteraceae* (1% bacteria). This phylum accounted for 11% of bacteria in sample R, but similarly to sample P, almost all classified sequences were affiliated with *Saccharopolyspora* sp. (7%), in addition of sequences related to the genera *Saccharomonospora*, *Propionibacterium* and *Brevibacterium* (each accounting for 1% of bacteria) being the last genera also identified in sample P.

The phylum *Firmicutes* constituted 26% and 1% of the total bacterial population in samples P and R, respectively (Supporting Information Fig. S2). In sample P most of the sequences affiliated with species of the genera *Staphylococcus* (10%), *Streptococcus* (3%), *Leuconostoc* (3%), *Lactobacillus* (2%) and *Gemella* (1%) as well as with members of the family *Carnobacteriaceae* (3%). In sample R, all members of *Firmicutes* were related to the genus *Staphylococcus* (Fig. 5).

The phylum *Proteobacteria* dominated in sample R (88% of bacteria, Supporting Information Fig. S2) being most sequences related to the *Gammaproteobacteria* class, especially to the genus *Pseudomonas* (69%), but also to the genera *Pantoea* (8%), *Acinetobacter* (7%) and *Enhydrobacter* (1%). Members of the *Alphaproteobacteria* and *Betaproteobacteria* were also detected in this sample but in proportions lower than 1% of the total bacterial population. This phylum was less dominant in sample P (30% of bacteria) and members of the *Gammaproteobacteria* contributed to 17% of bacteria with species mainly affiliated with the genera *Enhydrobacter* (10%) and *Acinetobacter* (2%), also identified in sample R, as well as *Haemophilus* (2%). The *Alphaproteobacteria* represented 11% of bacteria and comprised species of the genera *Brevundimonas* (9%) and *Neisseria* (2%).

Finally, members of the phylum *Bacteroidetes* were identified exclusively in the sample P (Supporting Information Fig. S2) and the sequences were related mainly to the genera *Porphyromonas*, *Prevotella*, *Sphingobacterium* and *Pedobacter*, being all of them in proportion <1% (data not shown).

#### Archaea

Archaea were only detected in the samples obtained from the Codex *Assemanianus* (Fig. 3). They accounted for a very low proportion of the total classified sequences (<0.5%), but it was possible to infer lower taxonomic levels (Supporting Information Fig. S3). Members of the family *Halobacteriaceae* were dominant in both samples, accounting for 80% and 100% of Archaea in P and R, respectively. Species of the genus *Halococcus* were identified in both samples (73% and 100% in P and R, respectively) while the genus *Halobacterium* (5%) could be detected only in sample P. In this last sample, members of the family *Methanobacteriaceae* were additionally detected (20%) with species of the genus *Metanobrevibacter*.

#### Eukaryotes

The proportion of Eukaryotes showed to be very low in the samples obtained from the *Liturgiarium Sinaiticum* (<1%). Nevertheless, it was still possible to infer lower taxonomic levels inside this domain (Supporting Information Fig. S4). Most of the sequences affiliated with the *Ascomycota* phylum (81% and 100% of eukaryotes in sample 5 and 6 respectively), namely with unclassified members of the family *Aspergillaceae*. The remaining 19% of the sequences retrieved from Eukaryotes in sample 5 were affiliated with the phylum *Apicomplexa*, being possible to identify species of the genus *Eimeria*, which were not identified in sample 6.

The proportion of Eukaryotes showed to be >1% in samples obtained from the Codex *Assemanianus* (1.5% and 1.2% of total sequences in samples P and R respectively). As observed in the *Liturgiarium Sinaiticum*, most of the sequences were affiliated with the *Ascomycota* phylum (78% and 99% of eukaryotes in samples P and R, respectively, Supporting Information Fig. S5) and were related to unclassified members of the family *Aspergillaceae* (49% and 99% of eukaryotes in P and R respectively). Sample P showed a higher diversity of *Ascomycota* and species of *Penicillium* and *Saccharomyces* (each 3%) as well as members of the family *Debaryomycetaceae* (24%) could be also identified. In addition, members of the phylum *Basidiomycota* were identified in sample P (22%) being all related to species of *Malassezia*. No members of *Basidiomycota* were found in sample R, but instead species of *Eimeria*, which belong to the phylum *Apicomplexa* (Supporting Information Fig. S5).

#### Viruses

Viruses were detected in all investigated samples (Fig. 3). Surprisingly, a relative proportion of the detected viruses showed to be related to RNA viruses. The proportion of DNA versus RNA viruses was shown to be different depending on the sample analysed (Supporting Information Figs. S6 and S7).

Sample 5 of the *Liturgiarium Sinaiticum* (Supporting Information Fig. S6) showed a relative high abundance of viruses, representing 3% of the total sequences. Most of them (66% of virus sequences) were related to DNA viruses, such as the dsDNA *Propionibacterium* phage‐P101A (61%) and the ssDNA Porcine bocavirus‐3 (5% of viruses). The remaining 34% of virus sequences detected in this sample affiliated with ssRNA viruses and proved to be related to the Jaagsiekte‐sheep retrovirus (25%), the Walrus calicivirus (3%) and the Dasheen mosaic‐virus (6%). The last RNA virus showed to be dominant in sample 6 (72% of viruses) in addition of the dsDNA Ahjdlikevirus (28%, Supporting Information Fig. S6).

The Codex *Assemanianus* P sample showed the highest relative abundance of viruses (15% of total sequences) as well as the highest diversity within this group (Supporting Information Fig. S7). Of these sequences, 95% were shown to be affiliated with dsDNA viruses and as observed in sample 5 of the *Liturgiarium Sinaiticum*, most of them were related to *Propionibacterium* phage‐P101A (49% of viruses). Additional dsDNA viruses, such as Merkel cell‐polyomavirus, *Mycobacterium* phage‐Angel, Betapapillomavirus‐1 and ‐4, Gammapapillomavirus‐1 and ‐9 and *Bombyx mori*‐nucleopolyhedrovirus were also detected in this sample with abundances >1% of the total viruses. The only ssRNA virus detected in this sample was affiliated with the Jaagsiekte‐sheep retrovirus (5% of viruses). In contrast, the sample R displayed a lower proportion of viruses (0.6% of all sequences) as well as a lower diversity, showing that all detected virus sequences affiliated with dsDNA viruses and were related to *Mycobacterium*‐phage‐DNAIII (86% of viruses) and *Bombyx mori*‐nucleopolyhedrovirus (14%).

## Discussion

The analysis of nucleic acids contained in historical parchments is a difficult matter due to the poor conservation of ancient DNA (aDNA) and the frequent external contamination of modern DNA (Vuissoz *et al*., 2007). The DNA of parchment can be degraded for a variety of reasons, including the chemical processes undergone during its manufacture, but also because of the antiquity of the parchment and the various alterations it may have suffered over the centuries because of human handling or improper conservation. Several scientific publications have addressed this issue and all of them point to the need of developing appropriate sampling methodologies, precise DNA extraction protocols for follow‐up analysis, as well as a correct DNA manipulation to avoid external contamination (Burger *et al*., 2000; Poulakakis *et al*., 2007; Campana *et al*., 2010; Pangallo *et al*., 2010; Teasdale *et al*., 2015, 2017). In addition, and considering the well‐known degradation of ancient DNA, previous studies have recommended the analysis of small DNA fragments for this kind of investigations (Burger *et al*., 2000; Campana *et al*., 2010). Most of the studies performed on parchment used PCR‐based assays for the sole purpose of recognizing and identifying the animal skin used for the manufacture of historical parchments (Poulakakis *et al*., 2007; Vuissoz *et al*., 2007; Pangallo *et al*., 2010). Nevertheless, several drawbacks have been documented with regard to PCR‐based assays, such as the control and estimation of contamination, the high proportion of chimeric PCR artefacts and the controversial results obtained using species‐specific primers (Campana *et al*., 2010; Pangallo *et al*., 2010). In addition, PCR favours the amplification of longer and less damaged DNA templates and therefore has a bias in favour of the contaminant over endogenous DNA (Teasdale *et al*., 2015). Recent studies have overcome this problem by performing next generation sequencing (NGS) analyses on parchment samples. In this context, it is important to note that metagenomics refers to untargeted sequencing analyses, the so‐called ‘shotgun approach’, which consists of sequencing the entire DNA library representing all the molecular components of a given sample (Teasdale *et al*., 2017). In contrast, a second procedure more commonly applied in microbial ecology studies through NGS analyses, focuses on specific conserved sequences such as ribosomal RNA genes (Piñar *et al*., 2019). The latter approach called the ‘amplicon sequencing approach’ has several advantages, such as less complexity of the data obtained, a larger number of sequences that can be assigned to a specific organism or a group of related organisms and, in addition, it can be applied for ‘target enrichment’ both during library preparation and after it for selecting particular DNA fragments of interest, especially when working with ancient DNA (Vai *et al*., 2017). The 16S rRNA and 18S rRNA genes, as well as the ITS regions, are the most common target regions when using NGS analyses to study the microbial structure of an environment, but because these regions are independently amplified they do not allow an overall estimation of the actual proportion of each of the life domains in the entire microbial community. However, despite this evidence, metagenomics (i.e. the shotgun sequencing approach) has been used sparingly in cultural heritage and, to our knowledge, specifically in a single study involving parchment materials (Teasdale *et al*., 2017).

Here, we decipher the biological information accumulated over more than 1000 years in two historical Slavonic parchment codices. The strategy used in this study followed a DNA extraction protocol based on the purification of short DNA fragments through silica membranes, which yielded a high efficiency in the recovery of total DNA (ancient and modern) from small pieces of parchment as well as from eraser fragments detached after non‐invasive sampling. In addition, we carried out a shotgun metagenomic analysis in order to avoid biases related to PCR‐based DNA research. All together enabled the simultaneous analysis of both, the raw material of the codex (i.e. the animal skins selected for manufacturing) and the microbiome colonizing the object, showing the real proportions of all life domains. The biological information obtained from the two codices investigated made it possible to determine the selection of the animal skins used for the manufacture of parchments, the probable vegetable origin of some inks, as well as the state of conservation and the latent risk of deterioration of these valuable objects. The following questions were addressed.

### What is the animal origin of the skins used for the manufacture of the investigated parchments?

The DNA sequences recovered from all investigated samples were aligned with the genomes of the main animal species used for the manufacture of parchments (cow, sheep, goat but also pig and human) in order to determine the affiliation of each of the investigated folios to one or another animal, but also to estimate the proportion of endogenous DNA (source species) retained in the different samples and their possible contamination with other animal species. In this study, the proportion of endogenous DNA ranged from 0.015% to 0.22% of total reads after passing the quality filters and after extracting the reads affiliated with human DNA (Table 2). This proportion is relatively low compared with the proportions of endogenous DNA obtained in the few comparable studies conducted on parchment to date. Teasdale *et al*. (2015) recovered 7.9%–9.4% of the endogenous sheep genome in two investigated codices, but it should be mentioned that the two scrolls were dated from the 17th and 18th centuries, so it is likely that the large percentage of endogenous DNA was facilitated by the younger age of the scrolls and the suitable conditions in which they were stored. In a subsequent study, Teasdale *et al*. (2017) aligned the DNA sequences obtained from the 1000‐year‐old bifolia of the York Gospels to the genome of the identified host species to estimate the proportion of endogenous DNA. They obtained an average percentage of endogenous DNA of 19.3% (range 0.7%–51.4%) in all samples. However, these assignments fell in the range of 0.2%–5.7% when filters for read mapping quality were applied. The loss of mapped reads is expected to occur after filtering, and the reduction suggests that there may be bias in the preservation and/or recovery of DNA sequences from the different manuscripts. This reflects the need to standardize protocols for comparing metagenomic studies, including DNA extraction and sequencing protocols, but even more importantly, data filtering, processing and statistical analysis.

Our results indicated that the codex *Liturgiarium Sinaiticum* was composed of folios made from skins of different animals (sheep and calf), what was usual at this time, where manufacturers used the skins they had accessible to them. This applies especially to the Sinai, where raw material has always been rare. In contrast, both of the samples analysed from the Codex *Assemanianus* were identified as sheep (Table 2). The presence of sequences representing multiple animal individuals has been already reported and can be attributed to some errors in sequencing and assembly due to aDNA degradation as well as to cross‐contamination in the parchment production process, during which multiple animal skins may have been washed, cured and depilated together (Campana *et al*., 2010; Teasdale *et al*., 2015). Sequences that aligned solely with the human genome were more abundant compared to those matching the host animals (ranging from 0.15% to 17% of total reads, Table 2), suggesting that they originate from handling of the parchments throughout their production and furthermore, their more recent origin due to an ongoing manipulation.

### What kind of information can the microbiome of parchments provide?

The information obtained from the microbiomes of the two investigated codices proved to be very interesting not only with regard to the conservation of the parchments. To the best of our knowledge, this is the first time that viruses are analysed as part of the microbiome of parchments, but it is important to clarify that the data obtained in this study regarding viruses are very preliminary and need more advanced bioinformatic analyses, such as genome assembly, to obtain definitive conclusions and not only initial hypotheses. However, these tentative hypotheses point to the direction of how to focus future studies on viruses in the emerging field of biocodicology. The presence of viruses was not dominant in some of the samples, but it revealed intriguing information that supports our findings about the animal origin of the skins and may even reveal the possible composition of some of the inks used to illustrate the codices.

In samples 5 and P the most dominant virus was *Propionibacterium* Phage P101A, which is related to the relative abundance of species of *Propionibacterium* in these two samples. However, an interesting finding was the detection of the Jaagsiekte sheep retrovirus (JSRV) (York and Querat, 2003). This beta‐retrovirus is the causative agent of the ovine pulmonary adenocarcinoma (OPA). The transmission of this virus occurs through aerosol spread between sheep. The exogenous infectious form of JSRV has an endogenous counterpart (enJSRVs), which is present in the genomes of all sheep, with about 27 copies per genome. Endogenous JSRV has multiple functions in the evolution of domestic sheep, by blocking the JSRV replication cycle and playing a key role in the development of the ovine embryo and placental morphogenesis (Arnaud *et al*., 2008). The detection of the JSRV in these two samples, through the DNA sequencing analyses performed in this study, seems to indicate that what has been detected are actually the endogenous counterpart sequences integrated in the sheep genome, which fully supports the affiliation of samples 5 and P with sheep. The absence of this virus in the microbiome of sample R, which was also affiliated with sheep, can be attributed to the less accurate recovery of genetic material from the eraser fragments. Finally, it should be noted that this virus was not detected in sample 6, which was affiliated with calf. The discovery of the ovine retrovirus in the samples manufactured from sheepskin is a very interesting result that could be used to study population genetics in parchment materials through more extensive bioinformatic analysis (Chessa *et al*., 2009), illustrating the multiple applications and possibilities offered by shotgun metagenomics in the field of biocodicology for future studies.

As mentioned in the Result section, a relative proportion of the detected viruses were identified as RNA viruses. Specifically, the two samples obtained from the *Liturgiarium Sinaiticum* showed a relative high abundance of the positive‐sense, single‐stranded RNA Dasheen mosaic virus (DsMV) (Supporting Information Fig. S6), which belongs to the genus *Potyvirus* (phytopathogenic viruses) and naturally infects the plant Taro (*Colocasia esculenta*) (Yusop *et al*., 2019). The detection of the DsMV in this Codex gives rise to some intriguing hypotheses. First, detecting this ssRNA virus in a DNA sequencing analysis may be explained by its integration into the genome of the host plant (Taro). All types of viruses can become endogenous by the integration of (partial) viral copy sequences into the genomes of various host organisms. These sequences are generally called endogenized viral elements (EVEs). To date, EVEs have been found originating from a diverse group of plant nuclear and cytoplasmic replicating DNA and RNA viruses, respectively (Takahashi *et al*., 2019). EVEs are commonly distinguished into two groups: those originating from retrovirus elements (endogenous pararetrovirus elements [EPREs]) and those containing any other plant virus sequence (endogenous non‐retrovirus elements [ENREs]). ENREs in host plant genomes are derived from segmented and rearranged viral sequences of dsRNA, ssDNA or ssRNA viruses (Chiba *et al*., 2011; Chu *et al*., 2014). The integration of potyvirus sequences into host plant genomes is well known (Takahashi *et al*., 2019) and may be the only plausible explanation for this finding. Unfortunately, we were unable to identify the DNA of Taro or any other related plant in the set of DNA sequences obtained from this Codex. However, if we track the presence of the DsMV and therefore its host plant (Taro) in this ancient Codex, a second hypothesis is raised through a bibliographic search. Taro is presumed to be one of the first cultivated plants, probably originating from Malaysian wetlands. The Taro was cultivated in humid tropical India 5000 B.C. and from India, it was transported westward to ancient Egypt, where Greek and Roman historians described it as an important crop (Grimaldi *et al*., 2018). The main use of this plant was the consumption of its edible leaves and corms, but it was also known for its high concentration of anthocyanin (Ghan *et al*., 1977). Bicchieri (2014) already reported that some inks used for parchment illustration were composed of anthocyanins extracted from different parts of plants, such as bark, leaves, fruits and seeds. These pigments were produced by precipitating the organic dye with an inert binder, usually a metallic salt. One hypothesis to explain the detection of the DsMV specifically in the *Liturgiarium Sinaiticum* could be that the Taro plant, very common in ancient Egypt, was used to extract anthocyanins for the local production of inks. In fact, previous studies conducted at the Academy of Fine Arts Vienna on this Codex, using X‐ray fluorescence analysis (XRF) in a non‐destructive and non‐invasive manner, led to the conclusion that an organic dye had been applied for the yellow and a blue pigment (Schreiner *et al*., 2008). As carbon, nitrogen or oxygen cannot be detected by XRF in air, no further conclusions could be drawn from the spectra. Therefore, no organic compounds could be specifically identified as anthocyanins. The identification of an organic compound would require original sample material, which cannot be taken from an object of the medieval period. Although only a hypothesis, the discovery of the Dasheen mosaic virus could indirectly support the previous theory, which some philologists suggest, that this codex was composed and written in the same place where it was found, namely in the monastery of St. Catherine in Sinai, Egypt (Miklas, 2000).

The eukaryotes represented only a small proportion of the parchment microbiomes (Fig. 3), which contrasts with previous studies in which molecular techniques based on the amplification of target regions (16S rRNA vs. ITS sequences) were applied to analyse the microbiomes of ancient manuscripts (Piñar *et al*., 2015a, b). However, this result confirms the most recent observations by Teasdale *et al*. (2017), where a low proportion of eukaryotes has also been observed in the microbiomes of several scrolls investigated by shotgun metagenomics, highlighting the greater precision of this strategy to study the real proportions of the different life domains in the microbiomes. Despite their low proportion, the eukaryotes provided some interesting information concerning the health status of the animals used to manufacture the parchments. Interestingly, some members of the *Apicomplexa* phylum, specifically of the genus *Eimeria*, were identified in samples 5 and R. The genus *Eimeria* comprises parasites that include several species able to cause coccidiosis in animals such as poultry, cattle and small ruminants, including sheep and goats (Chartier and Paraud, 2012). Coccidiosis often leads to diarrhoea, dehydration and weight loss. These combined factors can lead to poor growth and death of the animal, particularly among young lambs (Chartier and Paraud, 2012). The discovery of DNA traces of this parasite in these two sheep‐affiliated samples might indicate the poor health status of the animals used to manufacture the parchments.

Nevertheless, most of the information obtained from the microbiomes helped to understand the conservation status of the investigated parchments and gave an idea of their risk of deterioration. In this regard, most of the sequences affiliated with eukaryotes retrieved from all four samples showed to be related to *Ascomycota* (Supporting Information Figs. S4 and S5), namely to members of the *Aspergillaceae*. This finding is in line with previous studies. Members of this family, including species of *Penicillium* and *Aspergillus* were identified in all samples investigated in a survey dedicated to finding a microbial denominator corresponding to the measles‐like parchment discoloration phenomenon (Piñar *et al*., 2015a). These genera were also detected in several studies performed in deteriorated parchments (Polacheck *et al*., 1989; Pinzari *et al*., 2012; Troiano *et al*., 2014; Piñar *et al*., 2015b). Furthermore, members of the *Penicillium* genus have been proven to display a marked proteolytic activity on parchment (Kraková *et al*., 2012; Lech, 2016b). This fact indicates that there is a high latent risk of deterioration for the investigated parchments if the environmental conditions become suitable for the germination of these fungi.

Concerning bacteria, which represented the highest percentage of the microbiomes, the majority showed to be halotolerant/halophilic bacteria, grouping into different phylogenetic groups.

The *Liturgiarium Sinaiticum* showed a microbiome consisting of halotolerant/halophilic bacteria, mainly related to species of the genus *Nocardiopsis* in sample 5 and to species of *Gracilibacillus* in sample 6, being the last also present in sample 5. The presence of halotolerant/halophilic bacteria in parchment is a well‐known phenomenon and is related to the saline environment they present due to their manufacturing process (Piñar *et al*., 2015a, 2015b; Teasdale *et al*., 2017; Migliore *et al*., 2017, 2019). Parchment was prepared from animal skins with alkaline salts. Salting was performed in the early stages of manufacturing, immediately after skinning of the animal to inhibit microbial activity and to prevent the spoilage of the skins (Reed, 1975). The salts used were mainly sodium, potassium and ammonium chloride or sulfate in addition to lime and the procedure was done dry or in tanks, where the skins were immersed in brine for a few days to allow a deep penetration of salt ions into the skins (Migliore *et al*., 2017). Taking into account the repeated detection of halophilic and halotolerant organisms in parchments throughout independent studies, we can conclude that the salting process could act as an enrichment medium for these microorganisms, which could have been originally present in the salts used and transported to the skins during the salting process.

Nevertheless, during the manufacture of the parchment, the animal skins underwent further processes resulting in a product composed solely of the dermal skin layer, but the finished parchment also contained other compounds added during the production processes, such as salts and minerals (Bicchieri *et al*., 2019). For example, depilation was done by immersing animal skins in a solution of calcium hydroxide and further powders and pastes of calcium compounds were used to make the surface suitable for writing. Finally, additional lime, flour, egg whites and milk were added to the surface to make it softer. However, recipes for parchment preparation were different and introduced variations depending on each culture and geographical location (Bicchieri *et al*., 2019). Considering this laborious process, it is clear that there are traces of components that are likely to be biologically degraded, and ultimately contribute to the attack and degradation of the collagen fibres, which are the main component of parchment. Indeed, the dominant genera found in the *Liturgiarium Sinaiticum* (*Nocardiopsis* and *Gracilibacillus*) are known to have the capabilities to attack parchment. Species of the genus *Nocardiopsis* have been identified previously as part of the microbiota of deteriorated parchments (Piñar *et al*., 2015b). They produce a range of bioactive compounds and release new extracellular enzymes such as amylases, chitinases, cellulases, β‐glucanases, inulinases, xylanases and proteases. In particular, species of this genus secrete a number of alkaline proteases, milk clotting enzymes and keratinases, which help to use proteinaceous matter in nature (Bennur *et al*., 2015). In addition, an important characteristic of several species, specifically those detected in this study (*N. xinjiangensis* and *N. kunsanensis*) is their resistance to salt, alkalis and desiccation (Chun *et al*., 2000; Li *et al*., 2003).

Species of *Gracilibacillus* are also alkaliphilic and known to produce proteolytic enzymes able to hydrolyse gelatine (Wainø *et al*., 1999; Hirota *et al*., 2014), which is an irreversibly hydrolysed form of collagen. In addition, they have been reported to produce elastases (Varbanets *et al*., 2014), which break down elastin, an elastic fibre that together with collagen, determines the mechanical properties of tissues. Other species, such as *Yaniella halotolerans* and *Halobacillus* sp. were detected in the microbiome of the *Liturgiarium Sinaiticum*. Species of these two genera are halotolerant or halophilic and able to produce hydrolases (Müller and Oren, 2003; Li *et al*., 2004). Furthermore, species of *Halobacillus* have been previously detected in parchment samples showing biodeterioration (Piñar *et al*., 2015a).

The microbiomes derived from the two samples of the Codex *Assemanianus* showed differences in biodiversity. Sample P (piece of parchment) displayed more taxa compared to the sample R (eraser fragments), which is related to the type of sampling, making it evident that the information retrieved from a piece of parchment material, although very small, is more accurate than the information obtained when non‐invasive sampling is used. This fact further emphasizes the need to develop new accurate and non‐invasive sampling methods (Sterflinger *et al*., 2018). In this regard, it is important to note that in the eraser fragments, the largest proportion of bacterial sequences were affiliated with *Pseudomonas* species. This high proportion may be an artefact due to the sampling method, selecting those bacteria located in the more superficial layers of the parchment that are mere air contaminants. However, *Pseudomonas* species have been already described in the surface of deteriorated parchments (Piñar *et al*., 2015a) and have shown marked proteolytic activity (Kraková *et al*., 2012; Lech, 2016b).

Nevertheless, as observed previously in the other codex analysed in this study, the saline environment provided by the parchment influenced the composition of the microbiomes. The most important finding in both samples obtained from the Codex *Assemanianus* was the presence of *Saccharopolyspora* species in a relative high proportion. *Saccharopolyspora* has been described as a common microbial denominator detected in all investigated cases of a study devoted to investigate the causative agents of the so‐called ‘measles‐like’ parchment discoloration, consisting of purple spots associated with localized collagen damage and parchment degradation (Piñar *et al*., 2015a; Teasdale *et al*., 2017). In addition, some other Actinobacteria were identified in both samples in lower proportions, and belonged to the genera *Saccharomonospora* and *Actinopolyspora*, being species of both genera halophilic and halotolerant (Meklat *et al*., 2011). *Actinopolyspora* species have been already identified in parchments (Teasdale *et al*., 2017). Interestingly, the saline environment provided by this codex selected also halophilic members of the domain *Archaea*, being possible to identify species of the genera *Halococcus* and *Halobacterium*. Species of the genus *Halobacterium* have been previously identified in a single study performed on parchment (Migliore *et al*., 2019) but to our knowledge, this is the first time that *Halococcus* species have been identified in this material.

In addition to the dominant presence of halotolerant/halophilic microorganisms, it is important to highlight the detection of bacteria typical of the human microbiome in the four samples investigated. They corresponded to members of the genera *Propionibacterium* and *Staphylococcus* as well as the family *Dermabacteraceae*, which appeared with a higher or lower percentage in each of the samples. These bacteria have been previously detected in other studies carried out on parchments (Kraková *et al*., 2012; Piñar *et al*. 2015a; Lech, 2016b; Teasdale *et al*., 2017) and consider as human microbial markers due to the handling of the manuscripts along the centuries. However, the detection of these bacteria should be considered with caution, as some of them are potential pathogens. Some species of *Propionibacterium* and *Staphylococcus* but also of the *Dermabacteraceae* are common inhabitants of the human skin, but they can have pathogenic potential in opportunistic infections (Fitz‐Gibbon *et al*., 2013; Stackebrandt, 2014; Parlet *et al*. 2019). The genus *Brevibacterium* was detected in all samples, being impossible to infer the level of the species in most of them, with the exception of sample R, in which the species *B. linens* was identified. This species has been described as ubiquitous in human skin, where it causes foot odour, but also as a constituent of the microflora of surface smear‐ripened cheeses (Dixon, 1996). However, the most interesting feature is that this species is known to be halotolerant, capable of producing carotenoid pigments as well as a battery of proteolytic and peptidolytic enzymes (Rattray and Fox, 1999). All these characteristics indicate that this species poses a risk of damage to the parchment. The genus *Brevibacterium* has been previously identified in parchment studies (Kraková *et al*., 2012; Teasdale *et al*., 2017) but not the species *B. linens*.

Other potential pathogens were detected in only one of the codices. For instance, members of the genus *Bartonella* were identified only in the *Liturgiarium Sinaiticum*. Species of this genus are facultative intracellular parasites, which are considered especially important as opportunistic pathogens in humans and animals (Anderson and Neuman, 1997) and are transmitted by vectors such as ticks, fleas, sand flies, and mosquitoes. The genus *Megasphaera* was also identified in this codex (in sample 6 made of calf). Species of this genus have been isolated from various sources, including the rumen and faeces of cattle and other animals (Jeon *et al*., 2017). Finally, the genus *Rothia*, with the species *Rothia mucilaginosa*, is considered part of the normal microflora of the human mouth and the upper respiratory tract (Maraki and Papadakis, 2015).

## Conclusions

This study is an example to follow in the emerging field of biocodicology, demonstrating how DNA analysis and specifically shotgun metagenomics can help answer relevant questions that may contribute to a better understanding of the history of ancient objects. The biological information contained in ancient parchment codices provides and added historical value in the form of an individual ‘biological pedigree’.

The metagenomic strategy applied in this study allowed inferring the animal origin of the skins used in the manufacture of the two codices investigated, as well as to identify the microbiomes that colonize the surface of the parchments, which helped to determine their state of conservation and their latent risk of deterioration.

Our results showed that the saline environment provided by the parchments selects halophilic and halotolerant microorganisms, which are known to be responsible for the biodegradation and discoloration of parchment. Especially species of the genera *Nocardiopsis*, *Gracilibacillus* and *Saccharopolyspora* but also the members of the *Aspergillaceae* family detected in this study, all possess enzymatic capabilities for the biodeterioration of this material. It is important to highlight the relative abundance of microorganisms originating from the human skin microbiome, most probably related to the intensive manipulation of the manuscripts throughout the centuries, which should be taken with caution, as they can be potential pathogens. Finally, the preliminary results obtained on viruses in this study point to interesting hypotheses and the direction of how to focus future studies on viruses in the emerging field of biocodicology.

## Experimental procedures

### Objects investigated and sampling

Two valuable codices were investigated. The first one was the ‘*Liturgiarium (Missale) Sinaiticum*’ (Cod. Sin. Slav. 5/N), located in the library of the monastery of St. Catherine in Sinai, Egypt. Most probably, this codex was composed and written in the second quarter of the 11th Century in the same monastery. It is only fragmentarily preserved. While the bulk of the manuscript was found in 1975 in the Sinai, another fragment is kept in the Russian National Library, St. Petersburg. Two samples were obtained: sample 5 (Cod. Sin. Slav. 5 N, Folio 3) and sample 6 (Cod. Sin. Slav. 5 N, Fragment EDV 68). Both samples were detached pieces of the codex of approximately 2 mm^2^ × 5 mm^2^ that could not be reinserted in the folios in restoration campaigns (Fig. 1).

The second manuscript investigated was the Codex *Assemanianus* (Vat. Slav. 3), found in Jerusalem in the 18th Century, but written apparently at the beginning or in the second quarter of the 11th Century. The codex is currently located at the Vatican Apostolic Library, Rome, Italy. Two samples were collected: the first one referred as sample P (Vat‐Slav‐3P) was a detached piece of the codex of approximately 1 mm^2^ × 4 mm^2^. The second sample referred as sample R (Vat‐Slav‐Rb) was taken with an eraser, by rubbing the surface of the parchment and collecting the fragments detached from it in a sterile assay tube, as described by Fiddyment *et al*. (2015) (Fig. 2).

### DNA extraction, library preparation and sequencing

The extraction of DNA from the small pieces of parchments as well as from the eraser fragments collected from the Codex *Assemanianus* was performed in a separated laboratory dedicated only to the extraction of ancient DNA. DNA was extracted by a combination of the DNeasy Blood & Tissue Kit (Qiagene) followed by purification through the Qiagen MinElute cleanup (Qiagene). Briefly, the samples were transferred to a low binding Eppendorf tube and 180 μl buffer ATL (lysis buffer) + 20 μl Prot K were added, mixed by vortex and incubated o/n (~18 h) in a thermo‐block at 56°C and 600 rpm. On the next day, samples were centrifuged at 13 000 rpm for 5 min and the supernatant (180–200 μl) was transferred to a new low binding Eppendorf tube. Buffer PB from the Qiagen MinElute cleanup kit was added (5x Vol.) and the protocol was further applied following the recommendation of manufacturers. The DNA was eluted from the mini‐columns using 20 μl of preheated EB buffer (65°C). The quality and quantity of the resulting DNA was measured with a Nanodrop spectrophotometer and a Qubit fluorometer (using the dsDNA HS Assay Kit) respectively.

The DNA extracts obtained from all four samples were used for the creation of DNA libraries using the NEBNext Ultra II DNA Library Prep Kit for Illumina (New England Biolabs). Shotgun sequencing was performed using the Illumina HiSeq 2500 using 100 bp single reads for samples of both codices. Sequencing analyses were performed at the VBCF NGS Unit (www.viennabiocenter.org/facilities).

### Data analysis

Quality control of the reads was done with fastqc (Andrews, 2010). Cutadapt was used to trim adapter sequences (Martin, 2011). The cutadapt command line arguments for the four datasets are shown in the Supporting Information.

FastQ Screen was used to assess the origin of the parchment probes (Wingett and Andrews, 2018). The parchment metagenomes were assessed by first removing human and animal reads for each dataset. This was done for each experiment by filtering out the reads to the human and corresponding animal genomes with STAR (Dobin *et al*., 2013). The remaining reads were then processed with MetaphlAn2 in order to describe and represent the microbiomes of the parchment probes (Truong *et al*., 2015).

### Nucleotide sequence accession number

Raw sequencing data can be found in the BioProject PRJNA591636.

## Supporting information


**Supplementary Fig. S1.** Krona chart displaying the relative abundance of Bacteria identified in the *Liturgiarium Sinaiticum* at the genus level. Cutoff >1% of the total bacterial community. A) Sample 5 (Cod. Sin. Slav. 5 N, Folio 3) and B) sample 6 (Cod. Sin. Slav. 5 N, Fragment EDV 68).
**Supplementary Fig. S2.** Krona chart displaying the relative abundance of Bacteria identified in the Codex *Assemanianus* at the genus level. Cutoff >1% of the total bacterial community. A) Sample P (Vat‐Slav‐3P) and B) sample R (Vat‐Slav‐Rb).
**Supplementary Fig. S3.** Krona chart displaying the relative abundance of Archaea identified in the Codex *Assemanianus* at the genus level. Cutoff >1% of the total archaeal community. A) Sample P (Vat‐Slav‐3P) and B) sample R (Vat‐Slav‐Rb).
**Supplementary Fig. S4.** Krona chart displaying the relative abundance of Eukaryota identified in the *Liturgiarium Sinaiticum* at the family/genus level. Cutoff >1% of the total eukaryotic community. A) Sample 5 (Cod. Sin. Slav. 5 N, Folio 3) and B) sample 6 (Cod. Sin. Slav. 5 N, Fragment EDV 68).
**Supplementary Fig. S5.** Krona chart displaying the relative abundance of Eukaryota identified in the Codex *Assemanianus* at the family/genus level. Cutoff >1% of the total eukaryotic community. A) Sample P (Vat‐Slav‐3P) and B) sample R (Vat‐Slav‐Rb).
**Supplementary Fig. S6.** Krona chart displaying the relative abundance of viruses identified in the *Liturgiarium Sinaiticum*. Cutoff >1% of the total viruses. A) Sample 5 (Cod. Sin. Slav. 5 N, Folio 3) and B) sample 6 (Cod. Sin. Slav. 5 N, Fragment EDV 68).
**Supplementary Fig. S7.** Krona chart displaying the relative abundance of viruses identified in the Codex *Assemanianus*. Cutoff >1% of the total viruses. A) Sample P (Vat‐Slav‐3P) and B) sample R (Vat‐Slav‐Rb).
**Supplementary text.** The cutadapt command line arguments for the four datasets presented in this study.
