# Supplementary material for: Decoding the biological information contained in two ancient Slavonic parchment codices: an added historical value
Source: Environ Microbiol. 2020 May 29;22(8):3218–33. doi: 10.1111/1462-2920.15064 (PMC7687136; doi:10.1111/1462-2920.15064)
Supplement: Supplementary file 1 — Supplementary Fig. S1. Krona chart displaying the relative abundance of Bacteria identified in the Liturgiarium Sinaiticum at the genus level. Cutoff >1% of the total bacterial community. A) Sample 5 (Cod. Sin. Slav. 5 N, Folio 3) and B) sample 6 (Cod. Sin. Slav. 5 N, Fragment EDV 68). Supplementary Fig. S2. Krona chart displaying the relative abundance of Bacteria identified in the Codex Assemanianus at the genus level. Cutoff >1% of the total bacterial community. A) Sample P (Vat‐Slav‐3P) and B) sample R (Vat‐Slav‐Rb). Supplementary Fig. S3. Krona chart displaying the relative abundance of Archaea identified in the Codex Assemanianus at the genus level. Cutoff >1% of the total archaeal community. A) Sample P (Vat‐Slav‐3P) and B) sample R (Vat‐Slav‐Rb). Supplementary Fig. S4. Krona chart displaying the relative abundance of Eukaryota identified in the Liturgiarium Sinaiticum at the family/genus level. Cutoff >1% of the total eukaryotic community. A) Sample 5 (Cod. Sin. Slav. 5 N, Folio 3) and B) sample 6 (Cod. Sin. Slav. 5 N, Fragment EDV 68). Supplementary Fig. S5. Krona chart displaying the relative abundance of Eukaryota identified in the Codex Assemanianus at the family/genus level. Cutoff >1% of the total eukaryotic community. A) Sample P (Vat‐Slav‐3P) and B) sample R (Vat‐Slav‐Rb). Supplementary Fig. S6. Krona chart displaying the relative abundance of viruses identified in the Liturgiarium Sinaiticum. Cutoff >1% of the total viruses. A) Sample 5 (Cod. Sin. Slav. 5 N, Folio 3) and B) sample 6 (Cod. Sin. Slav. 5 N, Fragment EDV 68). Supplementary Fig. S7. Krona chart displaying the relative abundance of viruses identified in the Codex Assemanianus. Cutoff >1% of the total viruses. A) Sample P (Vat‐Slav‐3P) and B) sample R (Vat‐Slav‐Rb). Supplementary text. The cutadapt command line arguments for the four datasets presented in this study. [file EMI-22-3218-s001.pptx]

## Slide 1
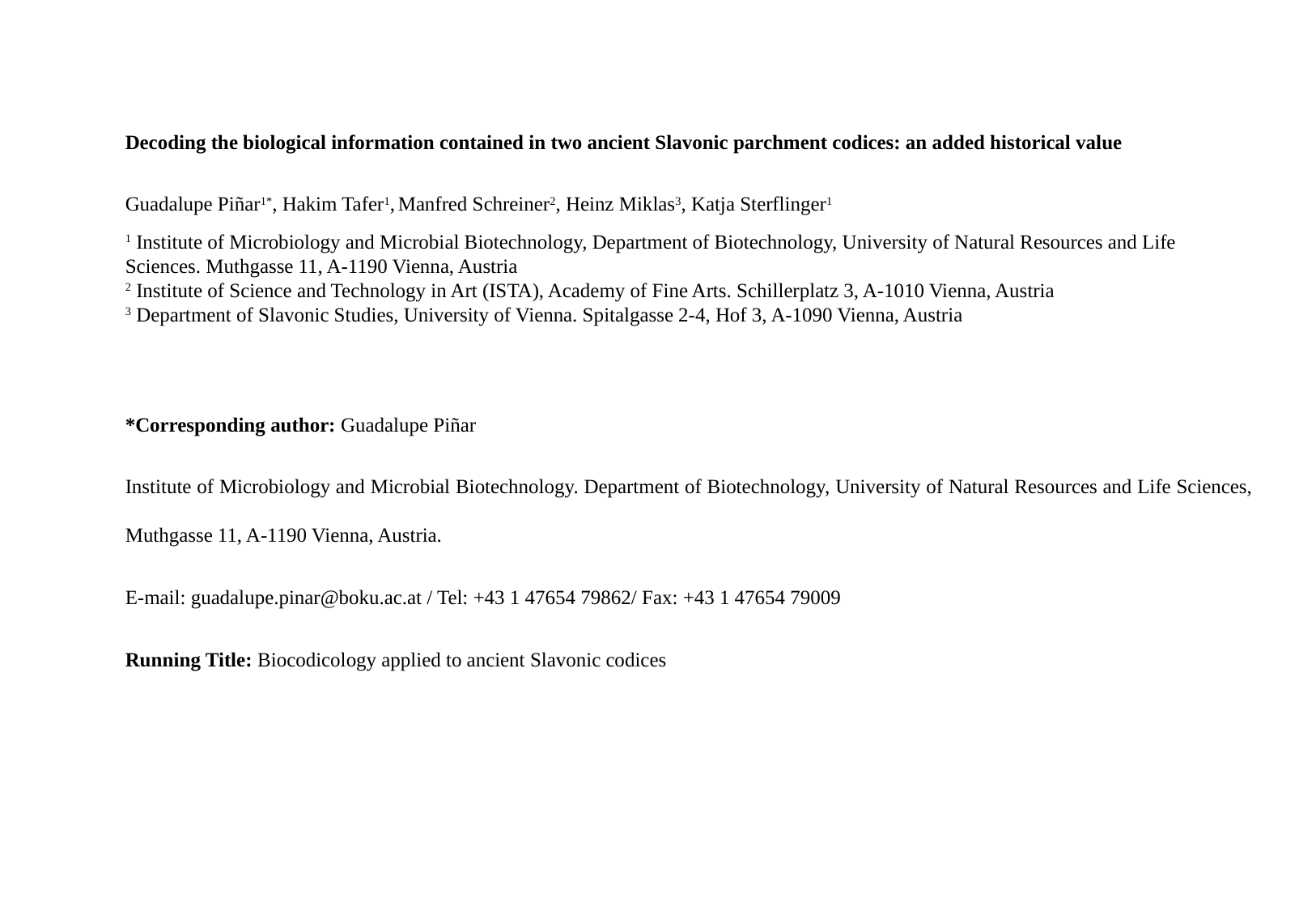

Decoding the biological information contained in two ancient Slavonic parchment codices: an added historical value
Guadalupe Piñar1*, Hakim Tafer1, Manfred Schreiner2, Heinz Miklas3, Katja Sterflinger1
1 Institute of Microbiology and Microbial Biotechnology, Department of Biotechnology, University of Natural Resources and Life Sciences. Muthgasse 11, A-1190 Vienna, Austria
2 Institute of Science and Technology in Art (ISTA), Academy of Fine Arts. Schillerplatz 3, A-1010 Vienna, Austria
3 Department of Slavonic Studies, University of Vienna. Spitalgasse 2-4, Hof 3, A-1090 Vienna, Austria
*Corresponding author: Guadalupe Piñar
Institute of Microbiology and Microbial Biotechnology. Department of Biotechnology, University of Natural Resources and Life Sciences, Muthgasse 11, A-1190 Vienna, Austria.
E-mail: guadalupe.pinar@boku.ac.at / Tel: +43 1 47654 79862/ Fax: +43 1 47654 79009
Running Title: Biocodicology applied to ancient Slavonic codices

## Slide 2
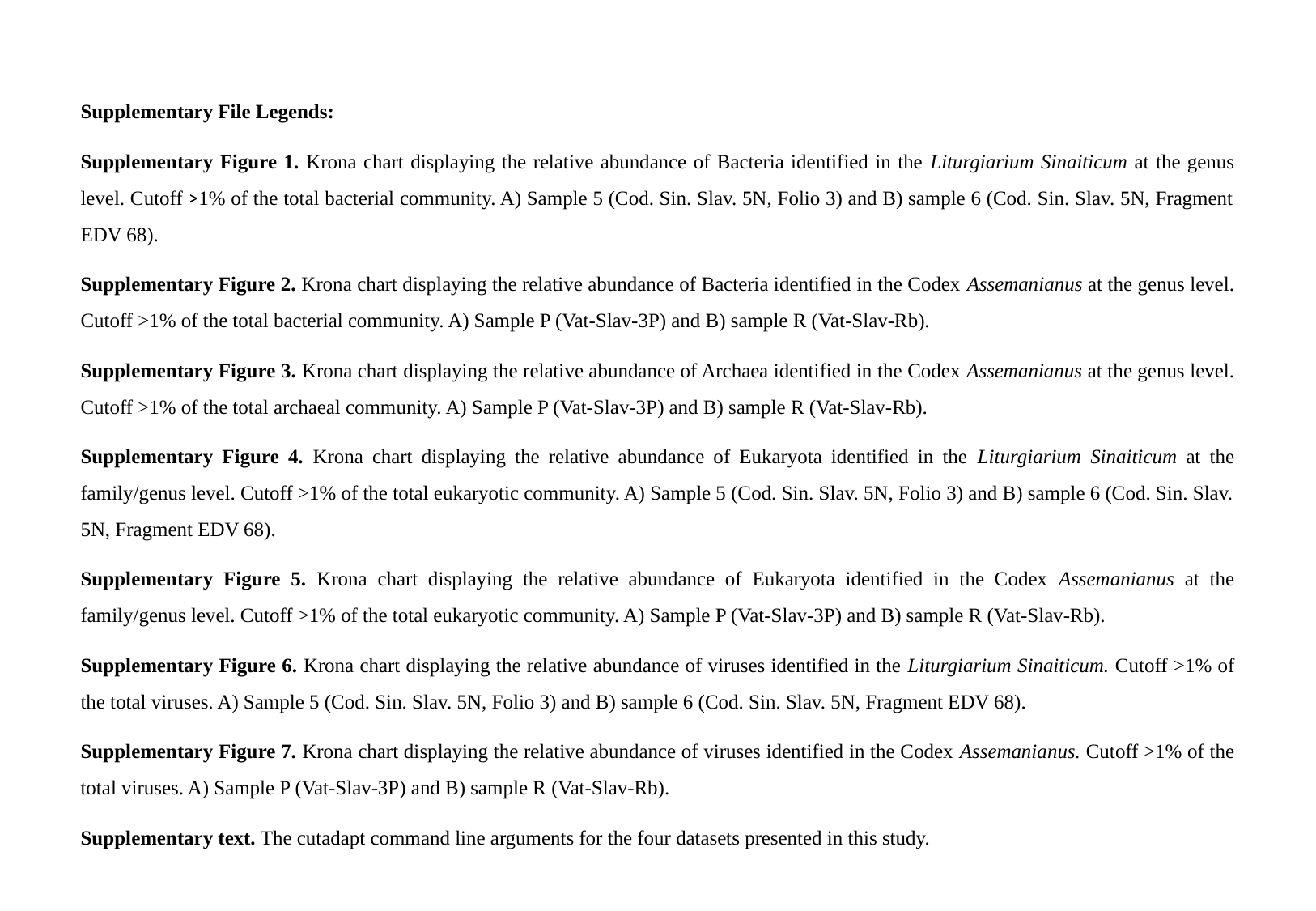

Supplementary File Legends:
Supplementary Figure 1. Krona chart displaying the relative abundance of Bacteria identified in the Liturgiarium Sinaiticum at the genus level. Cutoff ˃1% of the total bacterial community. A) Sample 5 (Cod. Sin. Slav. 5N, Folio 3) and B) sample 6 (Cod. Sin. Slav. 5N, Fragment EDV 68).
Supplementary Figure 2. Krona chart displaying the relative abundance of Bacteria identified in the Codex Assemanianus at the genus level. Cutoff ˃1% of the total bacterial community. A) Sample P (Vat-Slav-3P) and B) sample R (Vat-Slav-Rb).
Supplementary Figure 3. Krona chart displaying the relative abundance of Archaea identified in the Codex Assemanianus at the genus level. Cutoff ˃1% of the total archaeal community. A) Sample P (Vat-Slav-3P) and B) sample R (Vat-Slav-Rb).
Supplementary Figure 4. Krona chart displaying the relative abundance of Eukaryota identified in the Liturgiarium Sinaiticum at the family/genus level. Cutoff ˃1% of the total eukaryotic community. A) Sample 5 (Cod. Sin. Slav. 5N, Folio 3) and B) sample 6 (Cod. Sin. Slav. 5N, Fragment EDV 68).
Supplementary Figure 5. Krona chart displaying the relative abundance of Eukaryota identified in the Codex Assemanianus at the family/genus level. Cutoff ˃1% of the total eukaryotic community. A) Sample P (Vat-Slav-3P) and B) sample R (Vat-Slav-Rb).
Supplementary Figure 6. Krona chart displaying the relative abundance of viruses identified in the Liturgiarium Sinaiticum. Cutoff ˃1% of the total viruses. A) Sample 5 (Cod. Sin. Slav. 5N, Folio 3) and B) sample 6 (Cod. Sin. Slav. 5N, Fragment EDV 68).
Supplementary Figure 7. Krona chart displaying the relative abundance of viruses identified in the Codex Assemanianus. Cutoff ˃1% of the total viruses. A) Sample P (Vat-Slav-3P) and B) sample R (Vat-Slav-Rb).
Supplementary text. The cutadapt command line arguments for the four datasets presented in this study.

## Slide 3
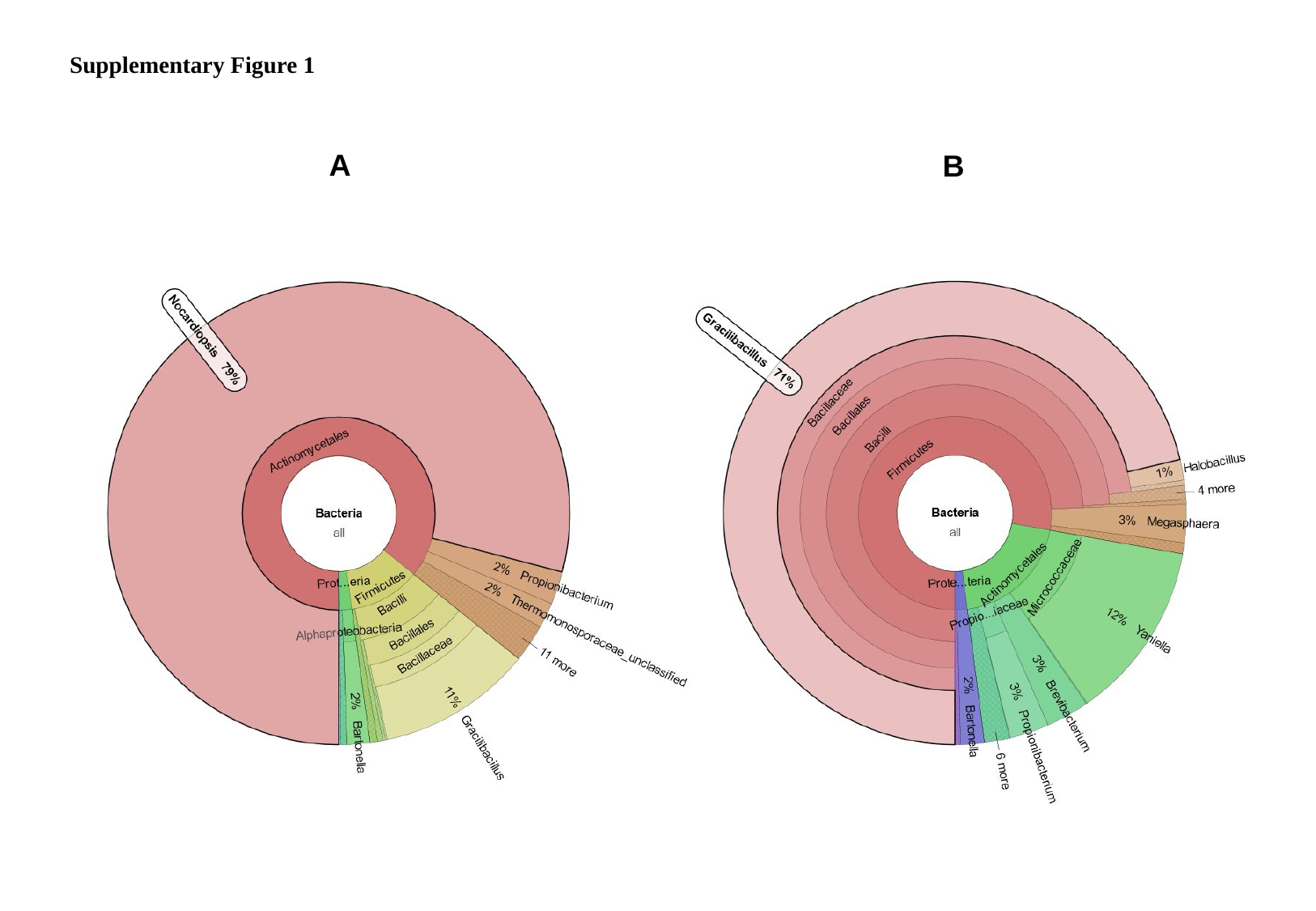

Supplementary Figure 1
A
B

## Slide 4
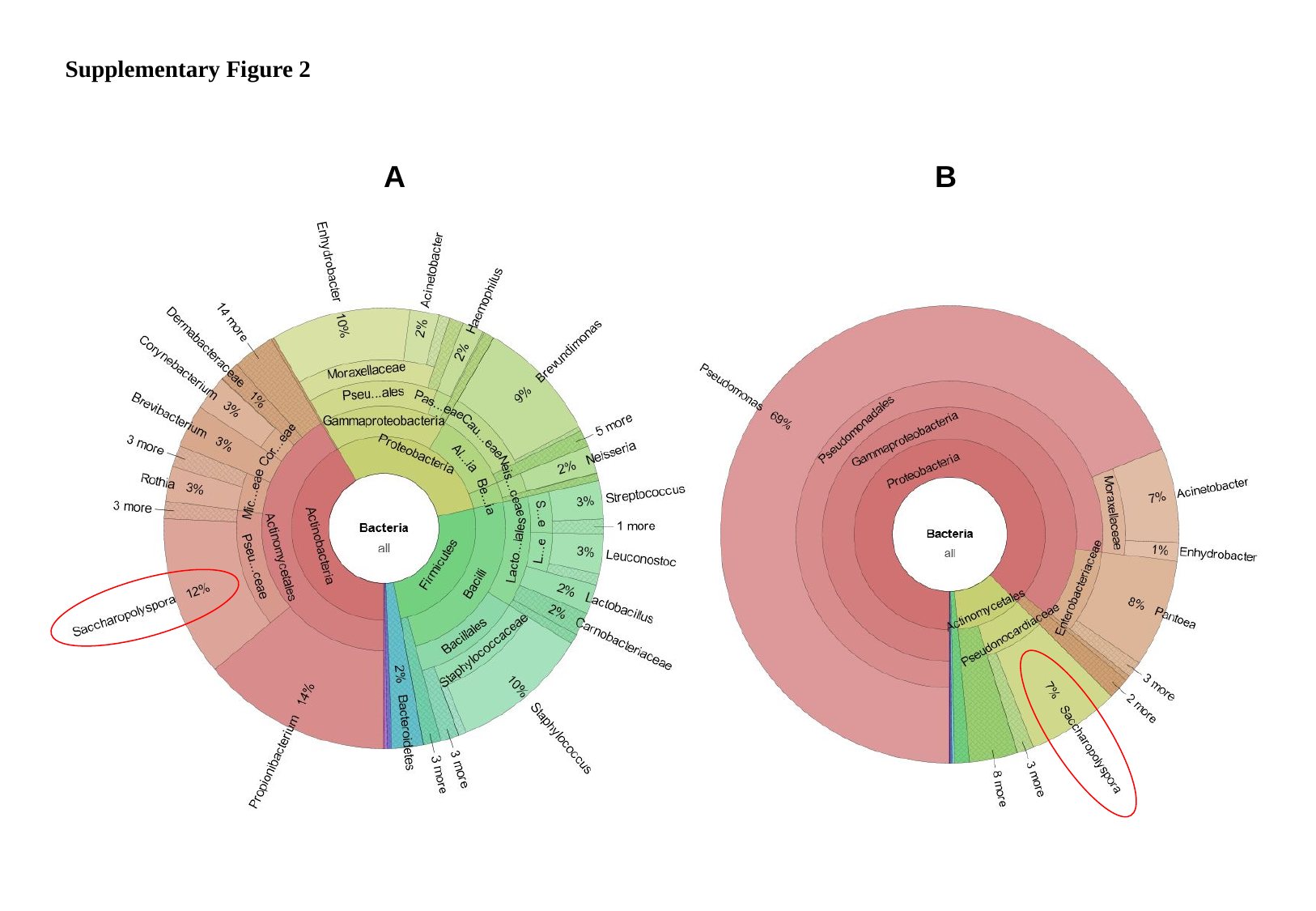

Supplementary Figure 2
A
B

## Slide 5
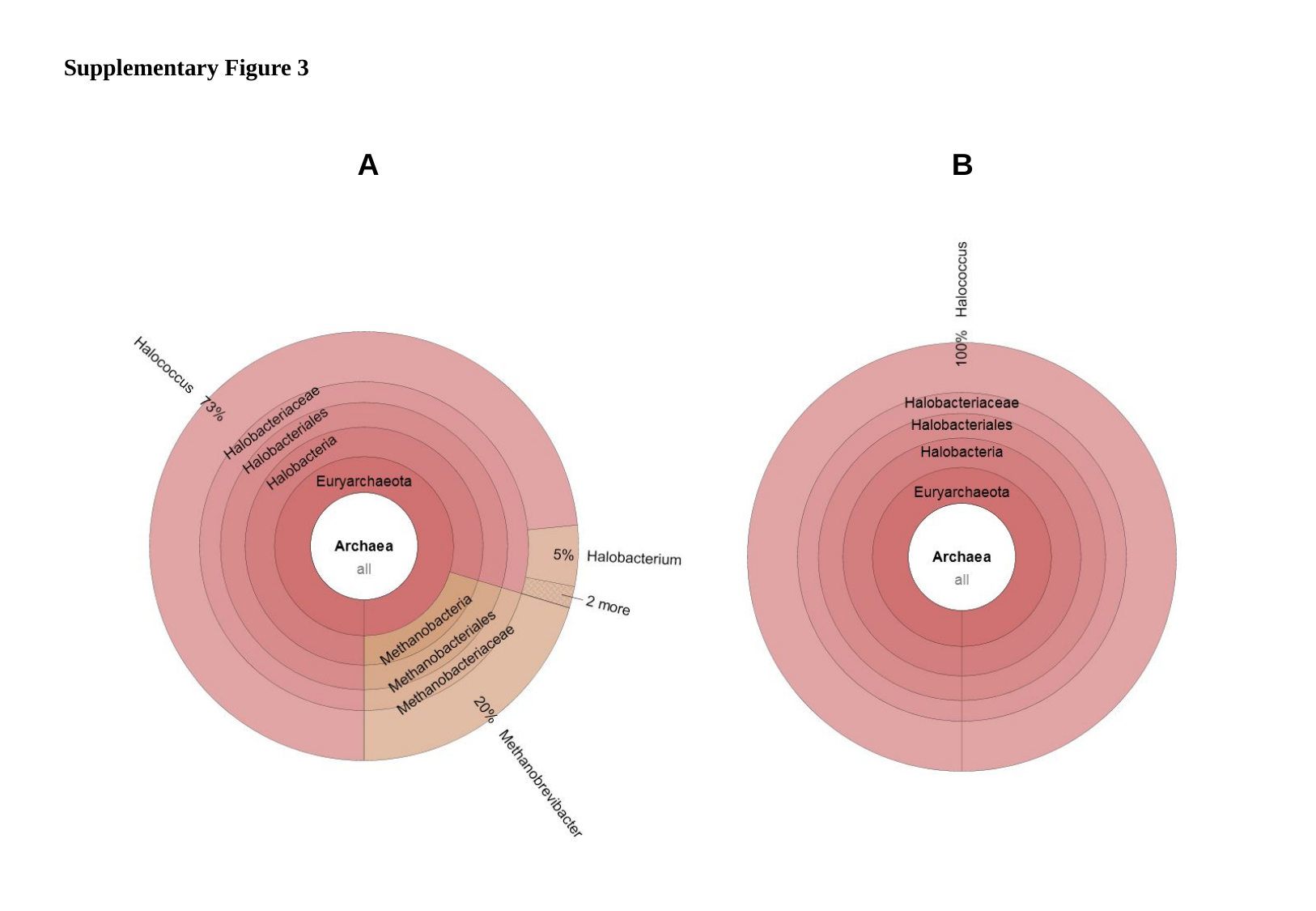

Supplementary Figure 3
A
B

## Slide 6
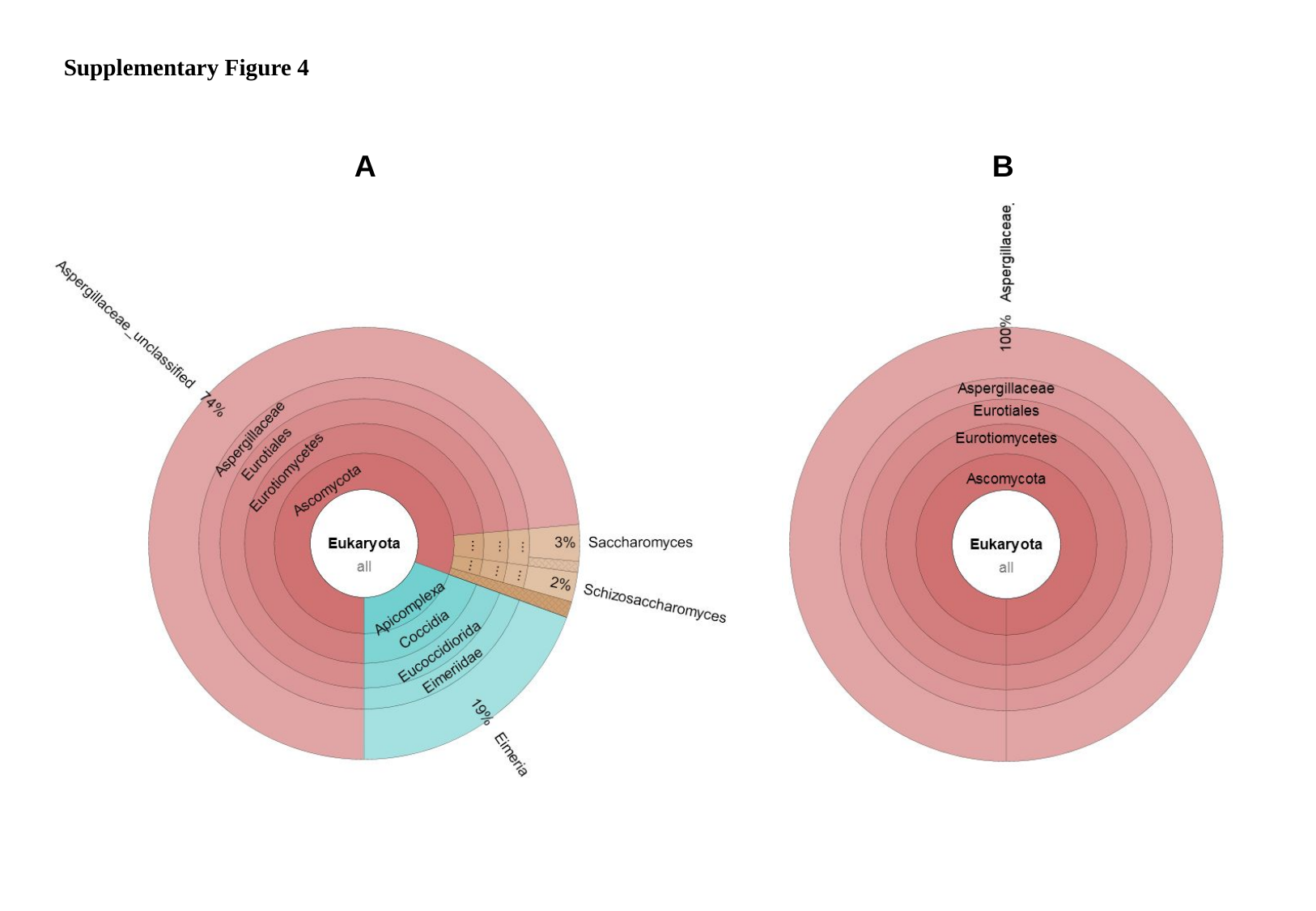

Supplementary Figure 4
A
B

## Slide 7
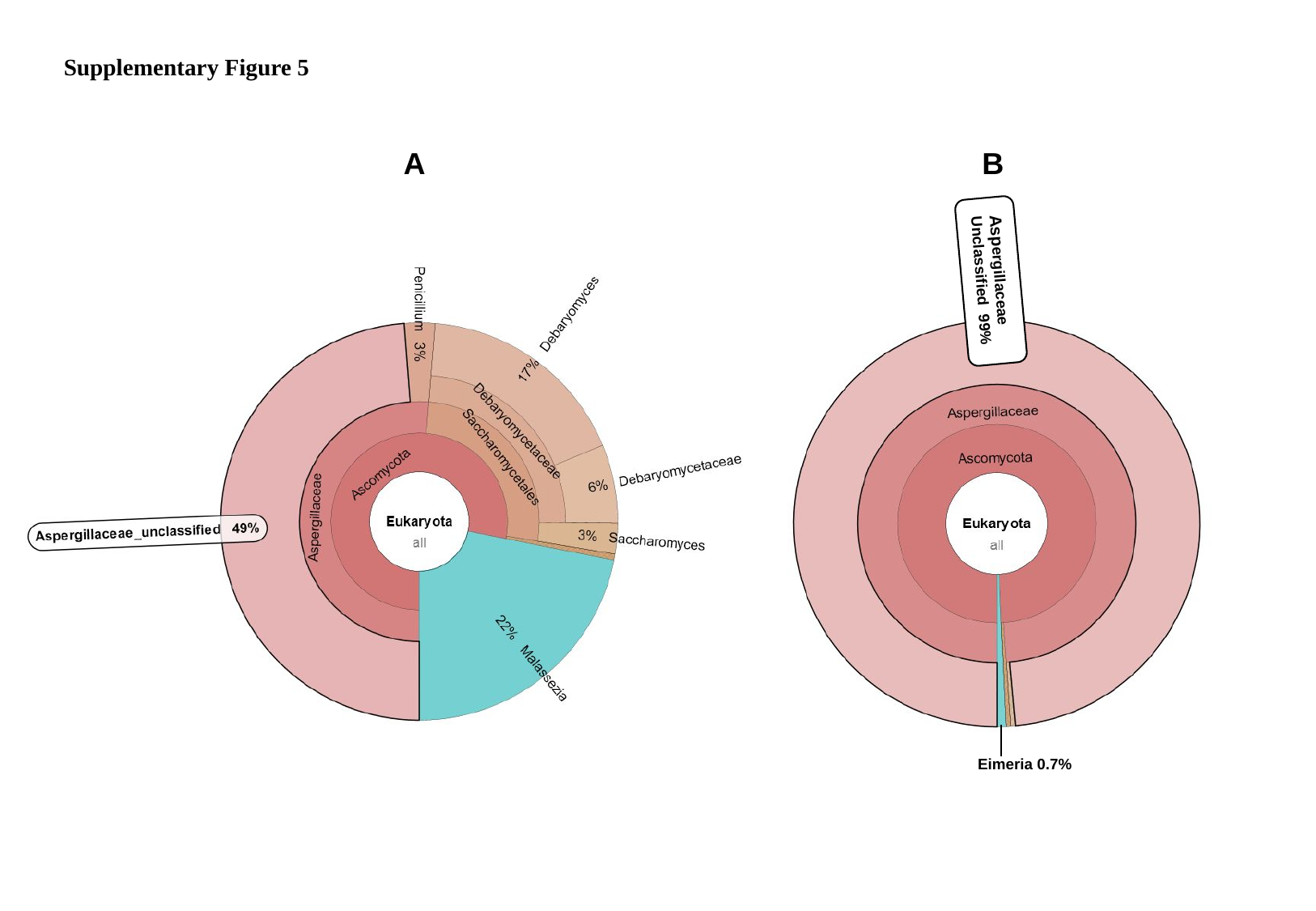

Supplementary Figure 5
A
B
Aspergillaceae
Unclassified 99%
Eimeria 0.7%

## Slide 8
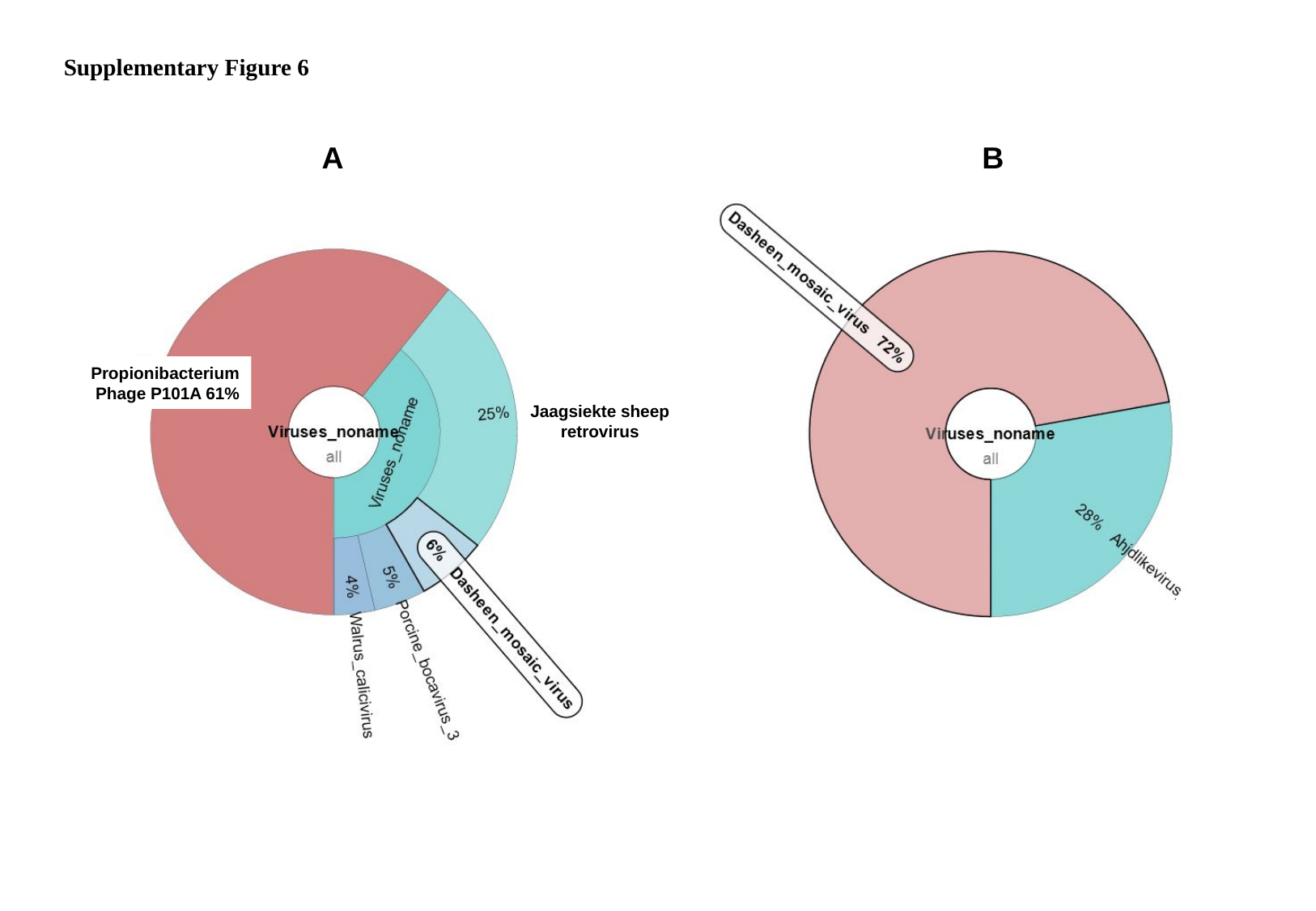

Supplementary Figure 6
A
B
Propionibacterium
Phage P101A 61%
Jaagsiekte sheep
retrovirus

## Slide 9
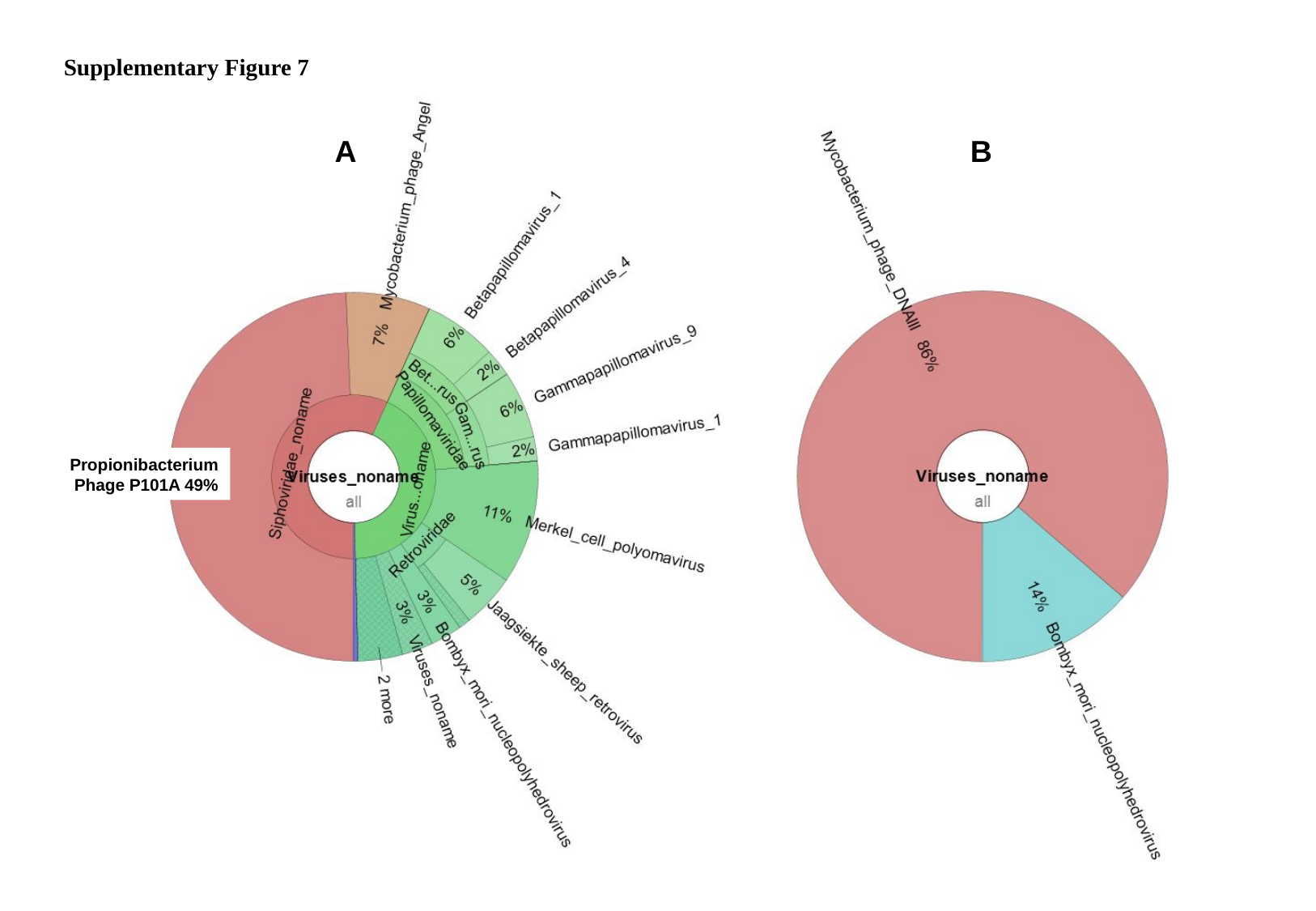

Supplementary Figure 7
Propionibacterium
Phage P101A 49%
A
B

## Slide 10
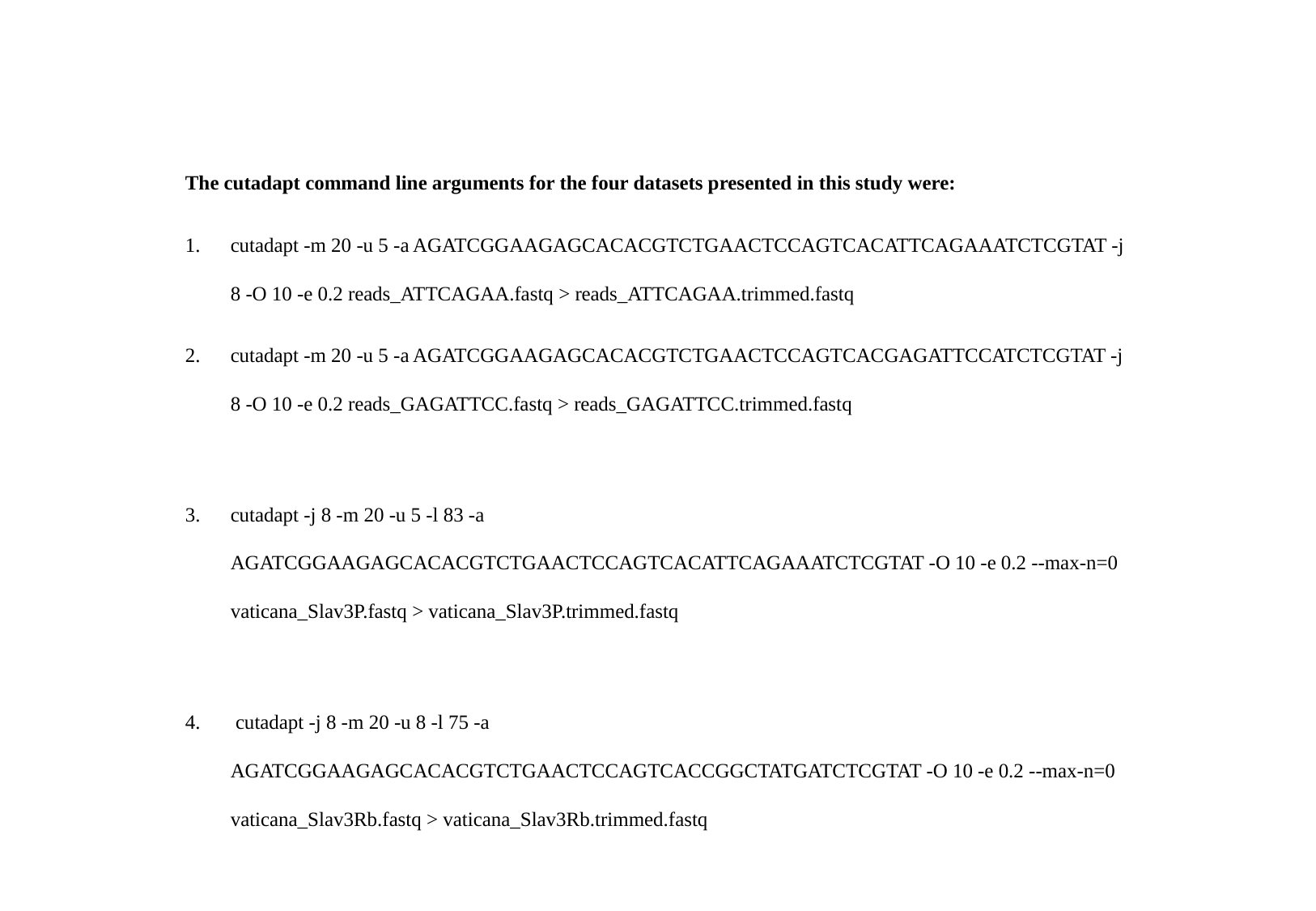

The cutadapt command line arguments for the four datasets presented in this study were:
cutadapt -m 20 -u 5 -a AGATCGGAAGAGCACACGTCTGAACTCCAGTCACATTCAGAAATCTCGTAT -j 8 -O 10 -e 0.2 reads_ATTCAGAA.fastq > reads_ATTCAGAA.trimmed.fastq
cutadapt -m 20 -u 5 -a AGATCGGAAGAGCACACGTCTGAACTCCAGTCACGAGATTCCATCTCGTAT -j 8 -O 10 -e 0.2 reads_GAGATTCC.fastq > reads_GAGATTCC.trimmed.fastq
cutadapt -j 8 -m 20 -u 5 -l 83 -a AGATCGGAAGAGCACACGTCTGAACTCCAGTCACATTCAGAAATCTCGTAT -O 10 -e 0.2 --max-n=0 vaticana_Slav3P.fastq > vaticana_Slav3P.trimmed.fastq
 cutadapt -j 8 -m 20 -u 8 -l 75 -a AGATCGGAAGAGCACACGTCTGAACTCCAGTCACCGGCTATGATCTCGTAT -O 10 -e 0.2 --max-n=0 vaticana_Slav3Rb.fastq > vaticana_Slav3Rb.trimmed.fastq
